# Inhibition of TBK1/IKKε Promotes Regeneration of Pancreatic β-cells

**DOI:** 10.1038/s41598-018-33875-0

**Published:** 2018-10-22

**Authors:** Jin Xu, Yun-Fang Jia, Subhasish Tapadar, Jessica D. Weaver, Idris O. Raji, Deeti J. Pithadia, Naureen Javeed, Andrés J. García, Doo-Sup Choi, Aleksey V. Matveyenko, Adegboyega K. Oyelere, Chong Hyun Shin

**Affiliations:** 1 School of Biological Sciences and the Parker H. Petit Institute for Bioengineering and Bioscience, Georgia Institute of Technology, Atlanta, GA 30332 USA; 2 Department of Molecular Pharmacology and Experimental Therapeutics, Mayo Clinic, Rochester, MN 55905 USA; 3 School of Chemistry and Biochemistry and the Parker H. Petit Institute for Bioengineering and Bioscience, Georgia Institute of Technology, Atlanta, GA 30332 USA; 4 Woodruff School of Mechanical Engineering and the Parker H. Petit Institute for Bioengineering and Bioscience, Georgia Institute of Technology, Atlanta, GA 30332 USA; 5 Department of Physiology and Biomedical Engineering, Mayo Clinic, Rochester, MN 55905 USA; 6 Present Address: Division of Biology and Biological Engineering, California Institute of Technology, Pasadena, CA 91125 USA

**Keywords:** Cell signalling, Small molecules

## Abstract

β-cell proliferation induction is a promising therapeutic strategy to restore β-cell mass. By screening small molecules in a transgenic zebrafish model of type 1 diabetes, we identified inhibitors of non-canonical IκB kinases (IKKs), TANK-binding kinase 1 (TBK1) and IκB kinase ε (IKKε), as enhancers of β-cell regeneration. The most potent β-cell regeneration enhancer was a cinnamic acid derivative (E)-3-(3-phenylbenzo[c]isoxazol-5-yl)acrylic acid (PIAA), which, acting through the cAMP-dependent protein kinase A (PKA), stimulated β-cell-specific proliferation by increasing cyclic AMP (cAMP) levels and mechanistic target of rapamycin (mTOR) activity. A combination of PIAA and cilostamide, an inhibitor of β-cell-enriched cAMP hydrolyzing enzyme phosphodiesterase (PDE) 3, enhanced β-cell proliferation, whereas overexpression of PDE3 blunted the mitogenic effect of PIAA in zebrafish. PIAA augmented proliferation of INS-1β-cells and β-cells in mammalian islets including human islets with elevation in cAMP levels and insulin secretion. PIAA improved glycemic control in streptozotocin (STZ)-induced diabetic mice with increases in β-cell proliferation, β-cell area, and insulin content in the pancreas. Collectively, these data reveal an evolutionarily conserved and critical role of TBK1/IKKε suppression in expanding functional β-cell mass.

## Introduction

Inflammation to islets has emerged as a key contributor to the loss of functional β-cell mass in both type 1 diabetes (T1DM) and type 2 diabetes (T2DM)^1,2^. In T1DM, β-cells are the target of an autoimmune assault. Chronic low-grade inflammation and activation of the immune system are major factors in obesity-induced insulin resistance and T2DM. Therefore, immunotherapies designed to block β-cell apoptosis may stand as a unifying target for diabetes treatment. Despite this rationale, the slow rate of β-cell regeneration in adult humans^3,4^ limits the efficacy of immune-intervention trials. Accordingly, among multiple small mitogenic molecules identified^5–18^, several of them have either not shown or shown minor functional effects in human β-cells^7–11^. Moreover, some of them displayed off-target effects^12,17,18^. Thus, identifying β-cell regenerating agents that specifically increase residual functional β-cells and coupling them with immunomodulators represent an auspicious treatment for T1DM and T2DM^19^.

Non-canonical IκB kinases (IKKs), TANK-binding kinase 1 (TBK1) and IKKε, have high sequence homology with comparable phosphorylation profiling of substrate(s)^20^. These kinases regulate inflammatory reactions primarily through their action on the interferon regulatory factor (IRF) pathway^21,22^. Independent of their role in acute immune responses, TBK1 and IKKε were shown to be induced in response to obesity-dependent inflammation and directly phosphorylate phosphodiesterase (PDE) 3B^23^, a major cyclic AMP (cAMP) hydrolyzing PDE isoform in adipocytes^24^. Consequently, pharmacological inhibition of TBK1/IKKε with amlexanox, a small molecule inhibitor of these kinases, increased cAMP levels in adipocytes^23^. This led to the secretion of interleukin-6 (IL-6) and the activation of the hepatic Signal Transducer and Activator of Transcription 3 (STAT3)^25^, resulting in weight loss and reduced hepatic gluconeogenesis in obese mice^26^. In addition, IKKε was shown to be among putative targets of diarylamide WS6, a small molecule that promoted human β-cell proliferation *in vitro*^9,11^. Despite these data suggesting a role for suppression of TBK1/IKKε in regulating glucose homeostasis and in expanding β-cells, the key question of how TBK1/IKKε function in β-cells remains elusive. Furthermore, validation of TBK1/IKKε inhibitors as mitogens for β-cells has yet to be reported.

Intracellular cAMP levels modulated by their rate of synthesis via adenylyl cyclase and their rate of degradation via PDEs^27^ are essential for β-cell replication, survival, and insulin secretion^10,28,29^. G-protein coupled receptors (GPCRs) adenosine receptor 2a (A2a) and 2b (A2b) activate Gαs and stimulate production of cAMP to increase β-cell proliferation during homeostatic control and regeneration of the β-cell mass^7,8^. Another G-protein coupled receptors GIP receptor (GIPR) and the GLP-1 receptor (GLP-1R) increase the level of cAMP in β-cells upon binding gastric inhibitory polypeptide (GIP) and glucagon-like peptide-1 (GLP-1), two primary incretin hormones, to exert their insulinotropic, anti-apoptotic, and proliferative effects^30–38^. Several of PDEs including PDE3B^39^ and PDE3A^40^ are highly expressed in β-cells. Inhibition of PDE3 with cilostamide reduced streptozotocin (STZ)-induced islet cell death in mice^40^ and augmented β-cell proliferation and regeneration in rat islets and zebrafish^7,10^. Intriguingly, cAMP-inducing β-adrenergic receptor (βAR) agonists have been shown to increase the mechanistic target of rapamycin (mTOR)-containing complex mTORC1 activity and *Uncoupling protein-1* (*Ucp1*) expression that critically contribute to white adipose browning^41,42^. In this study, cAMP-dependent protein kinase A (PKA) can directly phosphorylate mTOR, an evolutionarily conserved serine/threonine protein kinase pivotal for cellular growth and proliferation^43^, and the regulatory associated protein of mTOR^42^ (RAPTOR). This suggested βAR-cAMP-PKA-mTORC1 signaling pathway is novel and distinct from Insulin-Akt-mTORC1 signaling pathway^44–46^. Although TBK1/IKKε has demonstrated to blunt *Ucp1* expression in response to βAR agonists in 3T3-L1 adipocytes^23^, the signaling regulatory networks that link TBK1/IKKε, cAMP levels, and mTOR activity to proliferation and functional restoration of β-cells remain elusive.

In this study, through chemical screens using the zebrafish model of type 1 diabetes, we identified TBK1/IKKε inhibitors (TBK1/IKKε-Is) as enhancers of β-cell regeneration. Pharmacological and genetic functional analyses in zebrafish using the most promising hit-compound (E)-3-(3-**p**henylbenzo[c]**i**soxazol-5-yl)**a**crylic **a**cid (PIAA) indicated that suppression of TBK1/IKKε augments β-cell-specific proliferation by increasing cAMP levels and mTOR activity via PDE3. PIAA improved function and replication of mammalian β-cells including primary human β-cells. Furthermore, PIAA improved glycemic control and induced β-cell proliferation with increase in insulin content in the pancreas in streptozotocin (STZ)-induced diabetic mice.

## Results

### Chemical screens identify TBK1/IKKε inhibitors as enhancers of β-cell regeneration in zebrafish

**Figure 1:**
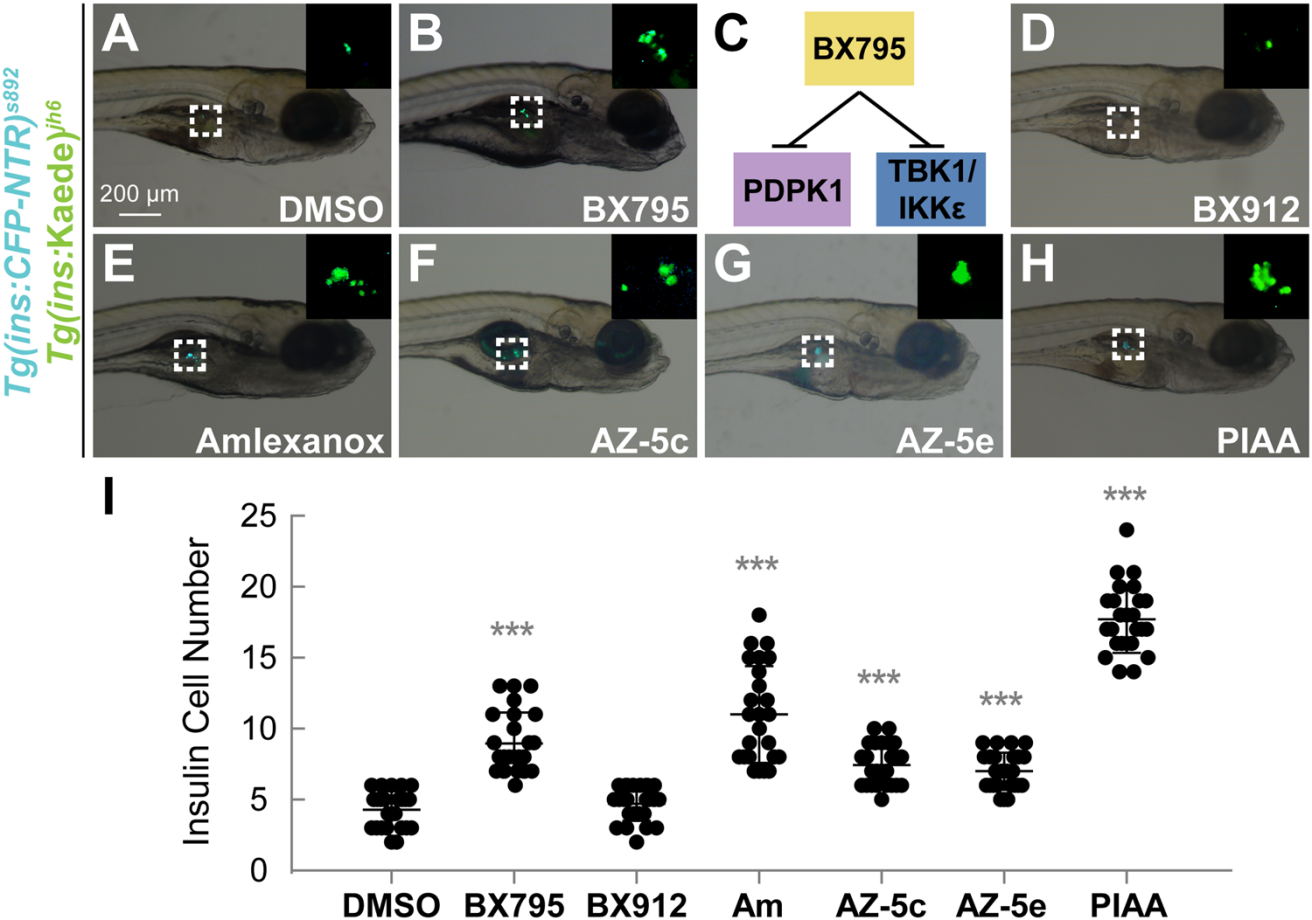
TBK1/IKKε inhibition augments β-cell regeneration in a zebrafish model of type 1 diabetes. (**A**,** B**,** D**–**H**) Bright-field images combined with fluorescent images showing the overall morphology and [*Tg*(*ins*:CFP-NTR)^*s892*^; *Tg*(*ins*:Kaede)^*jh6*^] expression (green) of larvae at 48 hpa treated with DMSO (**A**), BX795 (**B**), BX912 (**D**), amlexanox (**E**), AZ-5c (**F**), AZ-5e (**G**), and PIAA (**H**), respectively. While TBK1/IKKε-Is substantially expanded [*Tg*(*ins*:CFP-NTR)^*s892*^; *Tg*(*ins*:Kaede)^*jh6*^]-expressing cell population (white squares and insets) during regeneration (**B** and **E**–**H**) compared to DMSO (**A**), the PDPK1 inhibitor BX912 showed minimum effect (**D**). (**C**) BX795 is a dual inhibitor of PDPK1 and TBK1/IKKε. (**I**) Quantification of the number (mean ± SD) of total regenerated β-cells at 48 hpa (in A-B and D-H; 4.3 ± 1.3 (DMSO), 9.0 ± 2.2 (BX795), 4.7 ± 1.2 (BX912), 11.0 ± 3.4 (amlexanox), 7.4 ± 1.4 (AZ-5c), 7.0 ± 1.3 (AZ-5e), and 17.7 ± 2.4 (PIAA)). Cells in 20 planes of confocal images from 25 individual larvae were counted per condition. ***P < 0.001.

To identify bioactive compounds that facilitate pancreatic β-cell regeneration, we screened a library of 75 small molecules with well-characterized biological and pharmaceutical activity in a transgenic zebrafish model of type 1 diabetes. We used the *Tg*(*ins*:*CFP-NTR*)^*s892*^ line, in which β-cells are eradicated by nitroreductase (NTR), an enzyme that converts the chemical metronidazole (MTZ) to a DNA interstrand cross-linking agent^47,48^. To easily follow the ablation and regeneration of β-cells, we used an additional transgenic line, *Tg*(*ins:Kaede*)^*jh6*^, which expresses the brightly green fluorescent protein Kaede in β-cells^48^. [*Tg*(*ins*:*CFP-NTR*)^*s892*^; *Tg*(*ins:Kaede*)^*jh6*^] larvae were treated with MTZ at 3 days post-fertilization (dpf) for 24 hours to induce β-cell apoptosis, followed by washing out of MTZ (at 4 dpf, defined as 0 hours post-ablation (hpa)) and subsequent recovery in the presence or absence of chemical compounds for 48 hours (4–6 dpf, corresponding to 0–48 hpa). Using this system, we identified that the compound BX795 can approximately double the number of regenerated β-cells at 48 hpa (Fig. 1B, I). BX795 is a small molecule inhibitor that represses both 3-phosphoinositide-dependent kinase 1 (PDPK1) and non-canonical IKKs, TBK1 and IKKε^49^ (Fig. 1C). To determine which pathway’s suppression is primarily responsible for augmenting β-cell regeneration, we tested the potent PDPK1 inhibitor BX912^49^. BX912 caused minimal increase in β-cell regeneration (Fig. 1D, I).

**Figure 2:**
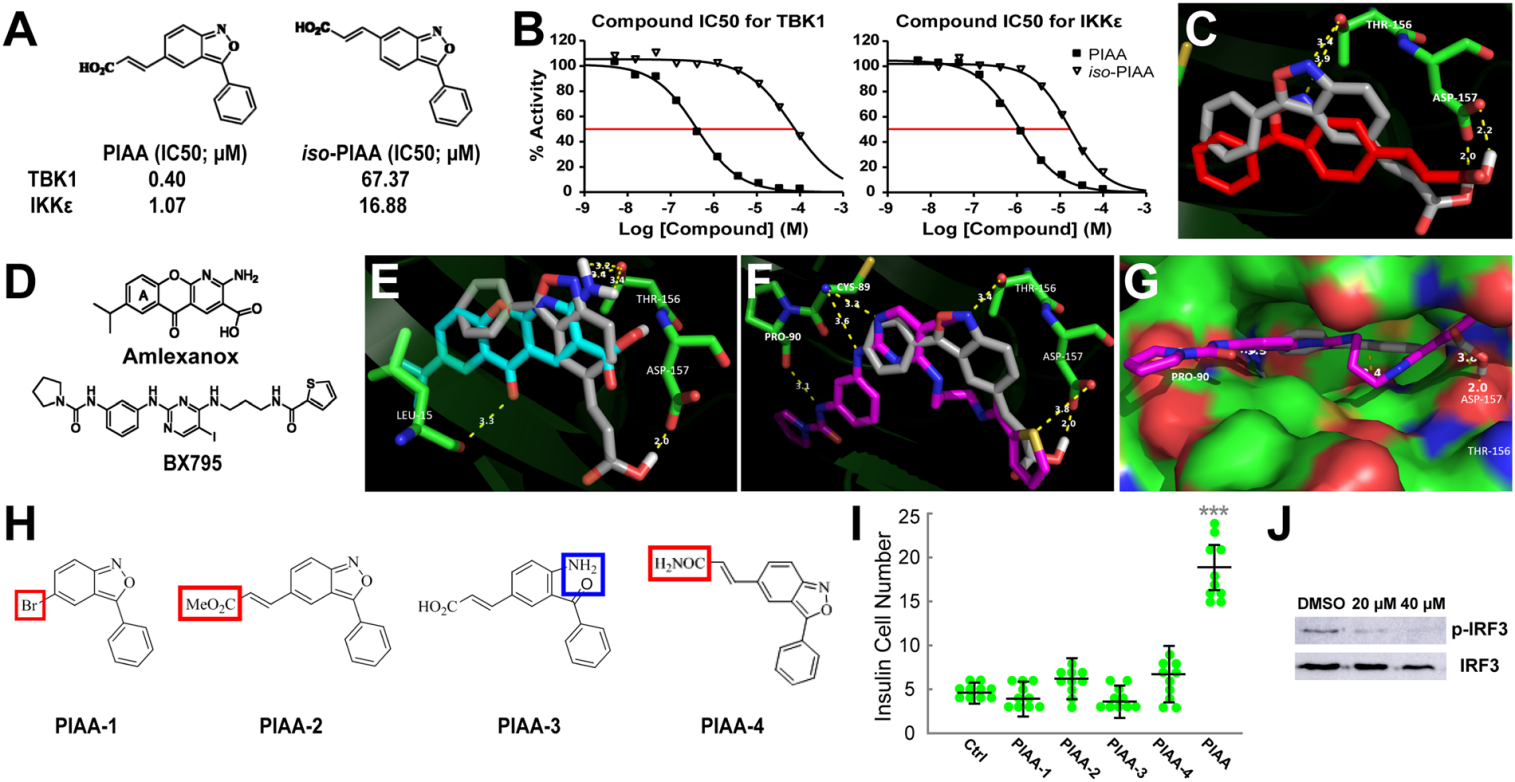
Kinase profiling, molecular docking, and structure-activity relationship analyses reveal selectivity of the TBK1/IKKε inhibitors. (**A**,** B**) Chemical structures and kinase profiling of PIAA and *iso*-PIAA. Dose responses of PIAA and *iso*-PIAA were generated to determine the potency of the inhibitors (IC_50_). (**C**) Ball and stick model of PIAA (grey) and *iso*-PIAA (red) docked into the binding pocket of TBK1. PIAA adapts a docked pose that has its isoxazole nitrogen and carboxylate moieties engaged in stronger interactions, relative to the same moieties on *iso*-PIAA, with THR-156 and ASP-157 at the active site of TBK1. (**D**) Chemical structures of amlexanox and BX795. (**E**–**G**) Molecular docking simulations showing interactions of TBK1/IKKε-Is and TBK1. Ball and stick model of PIAA (grey), amlexanox (blue), and BX795 (purple) docked into the binding pocket of TBK1 (**E** and **F**). Space filling model of PIAA (grey) and BX795 (purple) docked into the binding pocket of TBK1 (**G**). The core moieties of PIAA, amlexanox, and BX795 all bound within an unvaried region of the kinase domain of TBK1, while the urea moiety of BX795 extends toward the outer rim of the TBK1 kinase domain and interacts with the carbonyl group of Pro-90. Specifically, the carboxylate moieties of PIAA and amlexanox are placed next to the carboxylate side chain of ASP-157 buried in the TBK1 active site, forming a strong low-barrier H-bonding between these two carboxylate groups. (**H**) Chemical structures of four PIAA analogs. The moieties that were replaced and different from the original PIAA structure are marked in red (PIAA-1, PIAA-2, and PIAA-4) or blue (PIAA-3). (**I**) Quantification of the number (mean ± SD) of total regenerated β-cells at 48 hpa treated with DMSO, PIAA-1, PIAA-2, PIAA-3, PIAA-4, and PIAA, respectively (4.8 ± 0.8 (DMSO), 4.2 ± 1.3 (PIAA-1), 6.2 ± 1.3 (PIAA-2), 4.0 ± 1.2 (PIAA-3), 6.0 ± 2.2 (PIAA-4), and 18.6 ± 3.4 (PIAA)). Cells in 20 planes of confocal images from 10 individual larvae were counted per condition. ***P < 0.001. (**J**) Representative Western blot showing a PIAA dose-dependent decrease of pIRF3 levels in rat INS-1 cells.

Hence, we further tested the following biologically known as well as uncharacterized TBK1/IKKε inhibitors (TBK1/IKKε-Is): amlexanox^50^, azabenzimidazole (AZ) derivatives 5c and 5e^51^, and a cinnamic acid derivative (E)-3-(3-phenylbenzo[c]isoxazol-5-yl)acrylic acid (abbreviated as PIAA). PIAA was identified using a positional scanning peptide library (PSPL) technology^52^ and originally described as (E)-3-(3-phenylbenzo[c]isoxazol-6-yl)acrylic acid (*iso*-PIAA; Figs 2A–C and S1A, B; see details in Supplemental Experimental Procedures). Each of these compounds augmented the number of regenerated β-cells, with PIAA showing the highest efficiency (Fig. 1E–I). PIAA blocked the activity of IKKε with a half maximal inhibitory concentration of (IC50) approximately 1.07 μM and that of TBK1 at 0.4 μM (Fig. 2A, B). It did not block IKKα or IKKβ at these concentrations (IC50 of PIAA for IKKα: 47.2 μM; IKKβ: 50.5 μM). In addition, PIAA downregulated polyinosine:polycytidylic acid (poly I:C)-stimulated phosphorylation of interferon responsive factor-3 (IRF3), a substrate of TBK1/IKKε^53^, in INS-1 rat pancreatic β-cells (Figs 2J and S2). These results suggest that TBK1/IKKε inhibition, rather than PDPK1 repression, enhances β-cell regeneration.

To elucidate the interactions between TBK1/IKKε and inhibitors, we performed molecular docking simulations using the well-characterized TBK1 crystal structure in complex with BX795^54^ (PDB entry 4EUT). We observed that amlexanox and PIAA adapt docking poses that closely mimic the crystallographically obtained structure of BX795 by forming multiple hydrogen bonds (H-bonds) with key residues within the kinase domain of TBK1 (Fig. 2E–G). The core aromatic moieties, the carboxylate of amlexanox and PIAA, and the thiophene amide of BX795 all bound within an unvaried region of the kinase domain (Fig. 2E–G), while BX795, being a longer molecule, used its urea moiety to extend toward the outer rim of the kinase domain through interaction with the carbonyl group of Pro-90 (Fig. 2F, G). To corroborate the docking simulations, we further performed structure-activity relationship (SAR) studies of PIAA. We synthesized four analogs: PIAA-1 (an analog lacking the carboxylate group), PIAA-2 (a methyl ester analog of PIAA), PIAA-3 (an analog with an open isoxazolyl ring), and PIAA-4 (an amide analogue of PIAA) (Figs 2H and S1A). The carboxylate group and the intact isoxazolyl ring were both required in enhancing β-cell regeneration, since analogs lacking either group were inactive (Fig. 2I). Thus, the SAR data and modeling of TBK1 and inhibitor interactions delineate the molecular basis of the selectivity of the TBK1/IKKε-Is used in *in vivo* chemical screens. Taken together, these results indicate that suppression of TBK1/IKKε augments β-cell regeneration in the zebrafish model of type 1 diabetes.

### Repression of TBK1/IKKε increases β-cell regeneration by primarily promoting their proliferation

To exclude a substantial contribution of pre-existing β-cells to regeneration of β-cells, we converted the fluorescence of the Kaede protein from green to red by exposing the [*Tg*(*ins*:*CFP-NTR*)^*s892*^; *Tg*(*ins:Kaede*)^*jh6*^] larvae to UV light at 3 dpf immediately after MTZ treatment (Fig. S3A). We found that 48 hpa islets contained only unconverted green-only β-cells both in DMSO- or TBK1/IKKε-I-treated recovering larvae with a greater number of green-only β-cells in TBK1/IKKε-I-treated larvae (Fig. S3B). These results demonstrate that essentially all β-cells were ablated by MTZ treatment and that TBK1/IKKε-Is augment the number of the newly formed β-cells.

**Figure 3:**
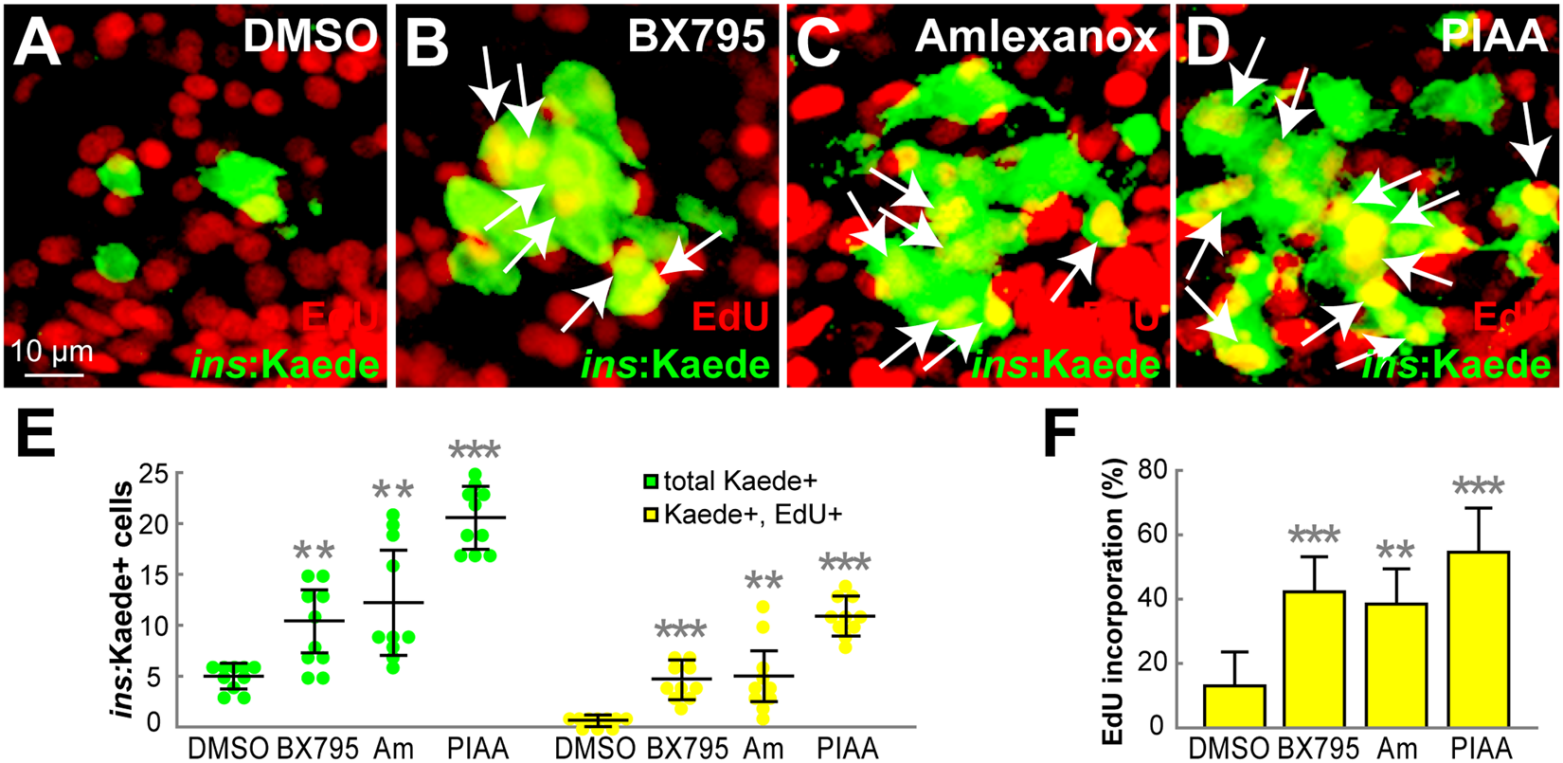
TBK1/IKKε inhibitors promote β-cell replication. (**A**–**D**) Confocal images of [*Tg*(*ins*:*CFP-NTR*)^*s892*^; *Tg*(*ins*:*Kaede*)^*jh6*^] larvae at 48 hpa, concurrently treated with EdU and DMSO (**A**), BX795 (**B**), amlexanox (**C**), or PIAA (**D**), respectively, from 0–48 hpa. The number of β-cells that incorporated EdU (white arrows) was substantially increased in TBK1/IKKε-I-treated recovering larvae (**B–D**) compared to DMSO-treated larvae (**A**). (**E**) Quantification of the number (mean ± SD) of total regenerated β-cells (green) and regenerated β-cells that incorporated EdU (yellow) at 48 hpa (in A-D; 5.0 ± 1.3 total regenerated β-cells, of which 0.7 ± 0.5 (DMSO), 11.0 ± 3.4, of which 4.6 ± 1.8 (BX795), 12.8 ± 4.8, of which 5.2 ± 2.8 (amlexanox), and 20.6 ± 3.1, of which 11.0 ± 1.9 (PIAA) incorporated EdU). (**F**) The percentage (mean ± SD) of regenerated β-cells that incorporated EdU at 48 hpa (in A-D; 13.0 ± 11.0% (DMSO), 42.0 ± 5.0% (BX795), 39.0 ± 7.0% (amlexanox), and 55.0 ± 14.0% (PIAA)). Cells in 20 planes of confocal images from 10 individual larvae were counted per condition. **P < 0.01; ***P < 0.001.

The TBK1/IKKε-I-induced increase in the number of newly regenerated β-cells could result from enhanced proliferation of β-cells, stimulation of neogenesis from non-β-cells, or both^55–58^. Hence, we determined the effect of TBK1/IKKε suppression on β-cell proliferation by performing cell cycle analysis with the replication marker 5-ethynyl-2′-deoxyuridine (EdU). [*Tg*(*ins*:*CFP-NTR*)^*s892*^; *Tg*(*ins:Kaede*)^*jh6*^] larvae were treated with MTZ from 3–4 dpf to ablate the β-cells, and subsequently treated with EdU and DMSO or TBK1/IKKε-Is for 48 hours (4–6 dpf, corresponding to 0–48 hpa). The number of β-cells that incorporated EdU was significantly greater in TBK1/IKKε-I-treated larvae than in DMSO-treated larvae (Fig. 3A–F).

**Figure 4:**
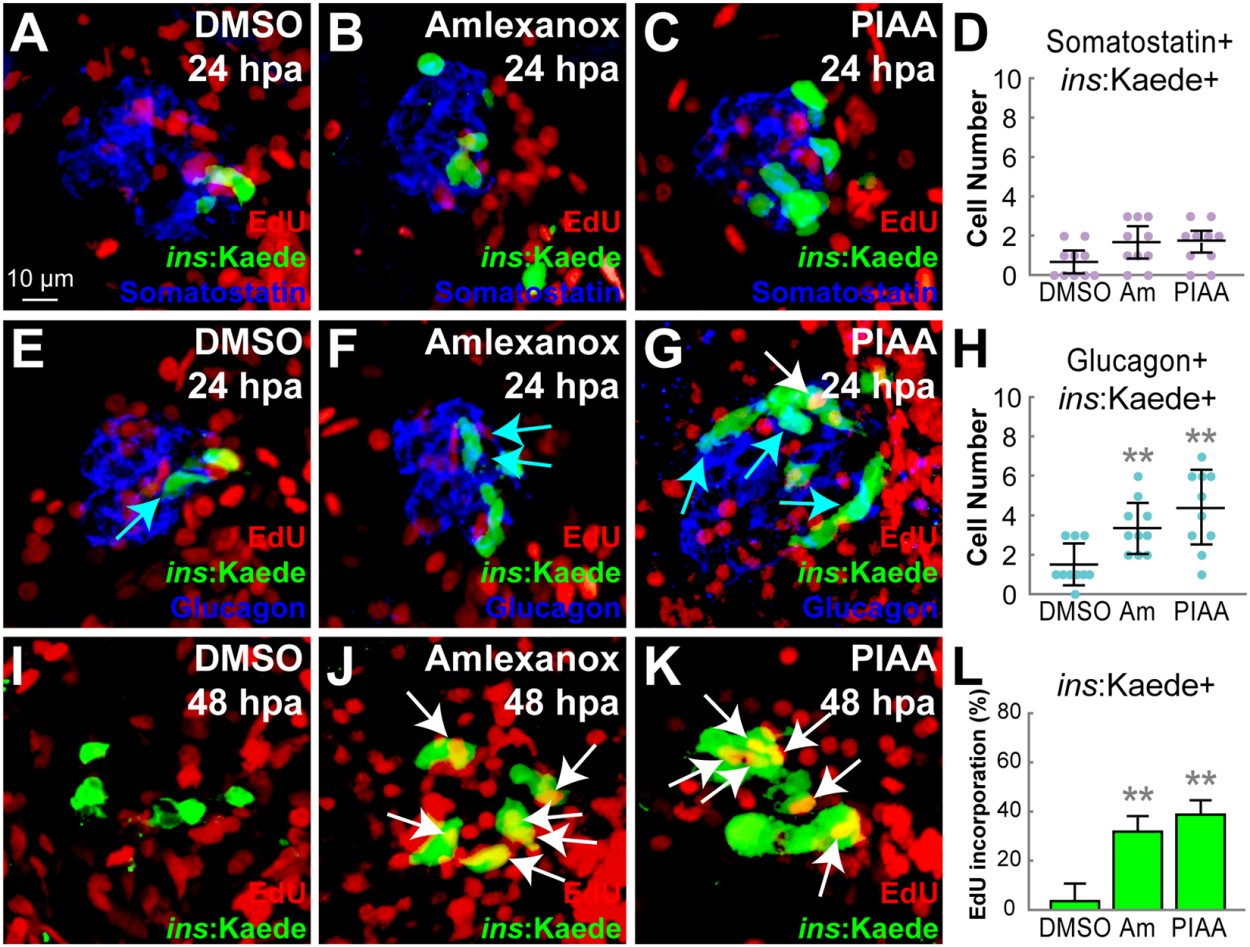
TBK1/IKKε inhibitors have modest effects on α-to-β-cell transdifferentiation but strongly enhance β-cell proliferation. (**A**–**C**) Confocal images of [*Tg*(*ins*:*CFP-NTR*)^*s892*^; *Tg*(*ins*:*Kaede*)^*jh6*^] larvae at 24 hpa, concurrently treated with EdU and DMSO (**A**), amlexanox (**B**), or PIAA (**C**), respectively, from 0–24 hpa, stained for Somatostatin (blue). (**D**) Quantification of the number (mean ± SD) of Insulin and Somatostatin-double positive cells at 24 hpa (in A-C; 0.7 ± 0.6 (DMSO), 1.7 ± 0.8 (amlexanox), and 1.8 ± 0.5 (PIAA)). (**E**–**G**) Confocal images of [*Tg*(*ins*:*CFP-NTR*)^*s892*^; *Tg*(*ins*:*Kaede*)^*jh6*^] larvae at 24 hpa, concurrently treated with EdU and DMSO (**E**), amlexanox (**F**), or PIAA (**G**), respectively, from 0–24 hpa, stained for Glucagon (blue). Note that the number of Insulin and Glucagon-double positive cells (blue arrows) was increased in TBK1/IKKε-I-treated recovering larvae (**F**,** G**) compared to DMSO-treated larvae (**E**). PIAA-treated larvae also showed an EdU-incorporated β-cell (white arrow) (**G**). (**H**) Quantification of the number (mean ± SD) of Insulin and Glucagon-double positive cells at 24 hpa (in E-G; 1.5 ± 1.1 (DMSO), 3.4 ± 1.3 (amlexanox), and 4.4 ± 1.9 (PIAA)). (**I**–**K**) Confocal images of [*Tg*(*ins*:*CFP-NTR*)^*s892*^; *Tg*(*ins*:*Kaede*)^*jh6*^] larvae at 48 hpa, concurrently treated with EdU and DMSO (**I**), amlexanox (**J**), or PIAA (**K**), respectively, from 24–48 hpa. The number of β-cells that incorporated EdU (white arrows) was significantly increased in TBK1/IKKε-I-treated recovering larvae (**J**,** K**) compared to DMSO-treated larvae (**I**). (**L**) The percentage (mean ± SD) of regenerated β-cells that incorporated EdU at 48 hpa (in I-K; 4.0 ± 7.0% (DMSO), 32.0 ± 6.0% (amlexanox), and 39.0 ± 6.0% (PIAA)). Cells in 20 planes of confocal images from 10 individual larvae were counted per condition. **P < 0.01.

A previous study showed that β-cell neogenesis occurs through both α-to-β-cell transdifferentiation and ductal progenitor-to-β-cell conversion during the initial stages of regeneration in zebrafish^59^. Administering TBK1/IKKε-Is, specifically amlexanox and PIAA, for 24 hours (from 4–5 dpf) rather than for 48 hours (from 4–6 dpf), caused minimal enhancement of the number of β-cells in/adjacent to the hepatopancreatic ductal (HPD) system and that of cells co-expressing Insulin and Somatostatin (Fig. 4A–D and data not shown). There was a slight increase in the number of cells that co-express Insulin and Glucagon (Fig. 4E–H). These results indicate that TBK1/IKKε repression does not primarily affect β-cell neogenesis. To further test whether these compounds increase proliferation of newly formed β-cells, we introduced a transitional day between β-cell ablation and TBK1/IKKε-I treatment. This time lag allowed MTZ-induced ablation to conclude and the default neogenesis to begin before the compounds were added. Amlexanox and PIAA both potently increased the number of EdU incorporated β-cells with the transitional day (Fig. 4I–L). Taken together, these data suggest that TBK1/IKKε suppression primarily enhances β-cell proliferation to promote regeneration of β-cells.

### TBK1/IKKε inhibition selectively accelerates proliferation of β-cells

**Figure 5:**
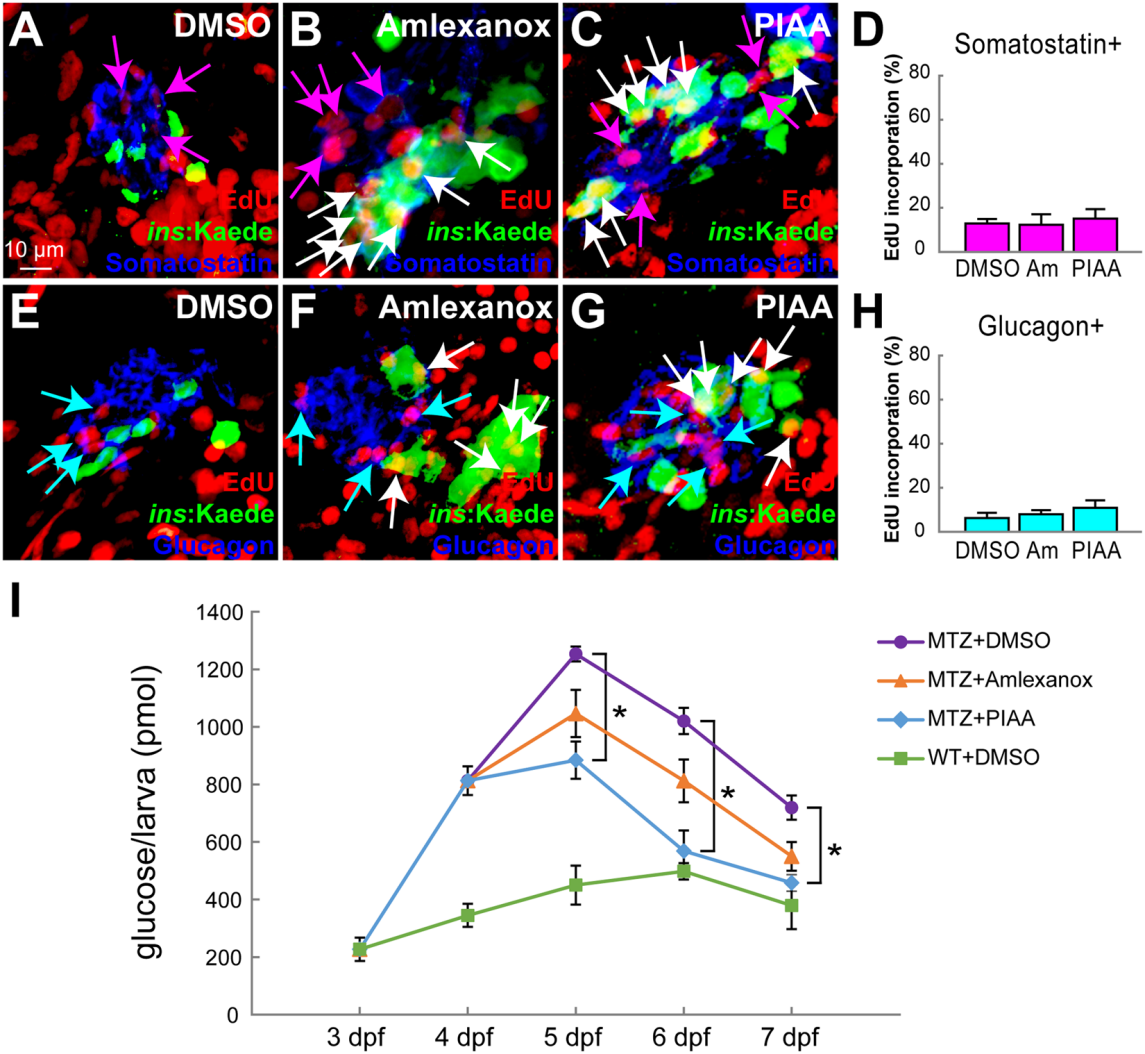
TBK1/IKKε inhibitors accelerate restoration of β-cell function by selectively increasing the number of β-cells. (**A**–**C**) Confocal images of [*Tg*(*ins*:*CFP-NTR*)^*s892*^; *Tg*(*ins*:*Kaede*)^*jh6*^] larvae at 48 hpa, concurrently treated with EdU and DMSO (**A**), amlexanox (**B**), or PIAA (**C**), respectively, from 0–48 hpa, stained for Somatostatin (blue). The number of Somatostatin-expressing δ-cells that incorporated EdU (purple arrows) did not increase in TBK1/IKKε-I-treated recovering larvae (**B**,** C**) compared to DMSO-treated larvae (**A**). (**D**) The percentage (mean ± SD) of δ-cells that incorporated EdU at 48 hpa (in A-C; 12.9 ± 2.0% (DMSO), 12.4 ± 4.7% (amlexanox), and 15.1 ± 4.3% (PIAA)). Cells in 20 planes of confocal images from 10 individual larvae were counted per condition. (**E**–**G**) Confocal images of [*Tg*(*ins*:*CFP-NTR*)^*s892*^; *Tg*(*ins*:*Kaede*)^*jh6*^] larvae at 48 hpa, concurrently treated with EdU and DMSO (**E**), amlexanox (**F**), or PIAA (**G**), respectively, from 0–48 hpa, stained for Glucagon (blue). The number of Glucagon-expressing α-cells that incorporated EdU (blue arrows) did not increase in TBK1/IKKε-I-treated recovering larvae (**F**,** G**) compared to DMSO-treated larvae (**E**). (**H**) The percentage (mean ± SD) of α-cells that incorporated EdU at 48 hpa (in E-G; 6.2 ± 2.4% (DMSO), 8.0 ± 1.9% (amlexanox), and 10.9 ± 3.4% (PIAA)). Cells in 20 planes of confocal images from 10 individual larvae were counted per condition. (**I**) Free-glucose levels (mean ± SD) during β-cell regeneration in non-ablated wild type, DMSO-treated recovering, and TBK1/IKKε-I-treated recovering larvae. At 7 dpf (equivalent to 72 hpa), free-glucose levels were significantly lower in PIAA-treated recovering larvae (blue line, 457.7 ± 28.8 pmol/larva) than in DMSO-treated larvae (purple line, 719.3 ± 42.2 pmol/larva). *P < 0.05. n = 30 larvae (3 pools of 10 larvae) per data point.

To determine whether TBK1/IKKε-Is increase proliferation of β-cells specifically or whether they trigger a general increase in cell proliferation, we assessed the replication rate of other pancreatic endocrine cells, specifically Somatostatin-producing δ-cells, Glucagon-producing α-cells, and liver cells. Inhibition of TBK1/IKKε led to minimal increase of EdU incorporation in δ-cells, whereas it enhanced proliferation of β-cells in the regenerating pancreas (Fig. 5A–D). TBK1/IKKε-Is also displayed no significant effects on α-cell replication (Fig. 5E–H). Furthermore, there was no considerable difference between DMSO- and TBK1/IKKε-I-treated larvae on the number of liver cells that incorporated EdU (Fig. S4A–D). A longer treatment with TBK1/IKKε-Is, for 96 hours after β-cell ablation, showed that TBK1/IKKε-Is did not lead to an overshoot in β-cell number (Fig. S4E–J and data not shown). These results suggest that suppression of TBK1/IKKε enhances β-cell proliferation during the most dynamic period of β-cell regeneration without inducing a general increase in proliferation of other cell types.

Next, we examined the ability of TBK1/IKKε-Is to restore normoglycemia. Free glucose levels were elevated after β-cell ablation but declined from 24–72 hpa (corresponding to 5–7 dpf) in DMSO- and TBK1/IKKε-I-treated larvae (Fig. 5I). Importantly, normal levels of free glucose were recovered significantly faster in TBK1/IKKε-I-treated, especially PIAA-treated, larvae than in DMSO-treated larvae (Fig. 5I). Altogether, these data suggest that inhibition of TBK1/IKKε induces selective increase in β-cell number that accelerates restoration of sufficient overall β-cell function.

### Repression of TBK1/IKKε enhances β-cell replication via cAMP-PKA-mTOR activation

To explore the mechanisms of how TBK1/IKKε suppression stimulates β-cell replication, we measured the regeneration efficiency of β-cells simultaneously treated with several β-cell replication-pathway inhibitors^8^ in the presence of TBK1/IKKε-Is, specifically PIAA. Whereas the phosphoinositide 3-kinase (PI3K) inhibitor LY294002 and Akt/Protein Kinase B (PKB) inhibitor MK2206 caused statistically significant increases in β-cell regeneration when combined with PIAA (Fig. S5K, L and S5N), the mechanistic target of rapamycin (mTOR) inhibitor rapamycin and the cAMP-dependent protein kinase A (PKA) inhibitor PKI-(6–22)-amide both suppressed PIAA-mediated β-cell regeneration (Fig. S5I, J and S5N). We observed the same effect when each of LY294002 and MK2206 was tested alone (Fig. S5E, F and S5M). These results implicate that cAMP-PKA and mTOR signaling mediate the β-cell regeneration response to TBK1/IKKε-Is (Fig. S5O) and that PI3K-Akt signaling, cooperatively with TBK1/IKKε signaling, may play a repressive role in β-cell regeneration (Fig. S5O).

**Figure 6:**
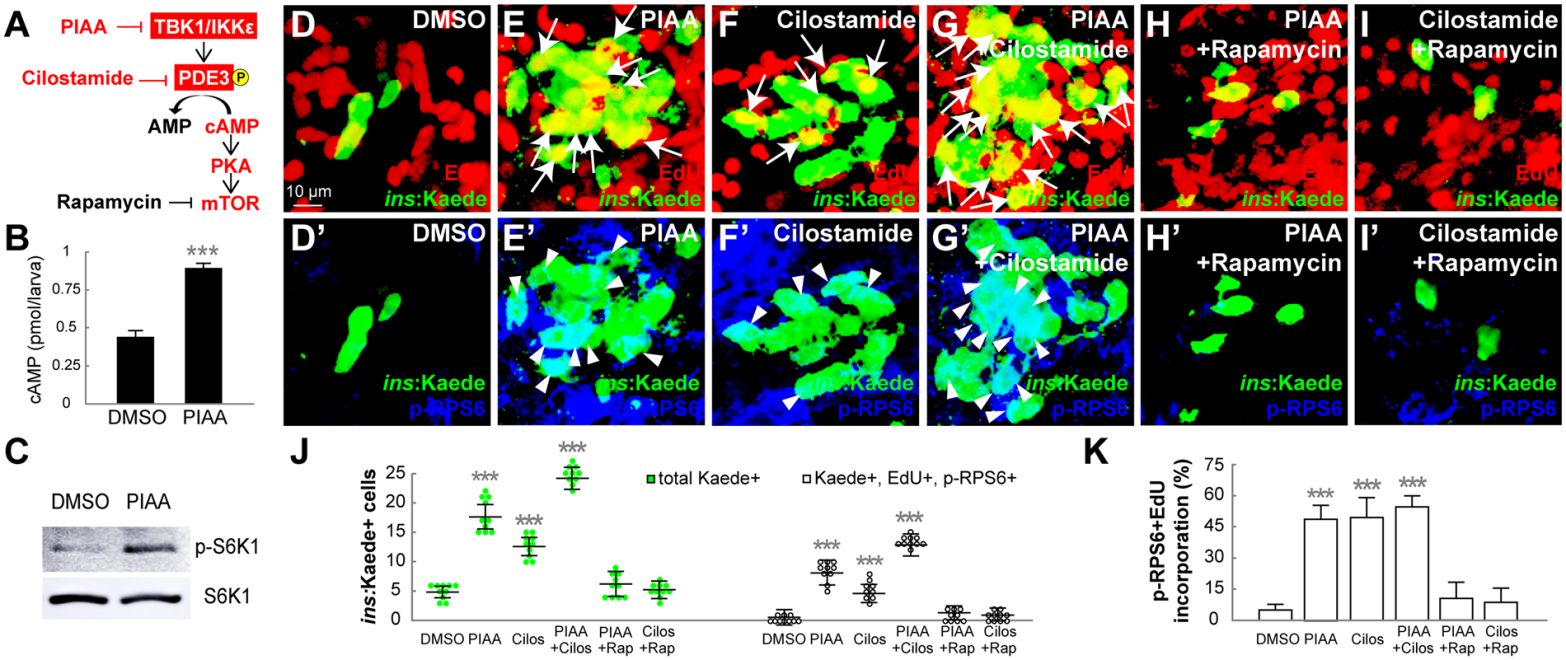
Suppression of the TBK1/IKKε-PDE3 signaling axis promotes β-cell proliferation by increasing cAMP levels and mTOR activity. (**A**) Schematic of the TBK1/IKKε-PDE3 signaling that modulates cAMP-PKA-mTOR pathway. The sites of inhibition by PIAA and cilostamide are shown in red. (**B**) Quantification of cAMP levels (mean ± SD) at 48 hpa (0.4 ± 0.1 pmol/larva (DMSO) and 0.9 ± 0.0 pmol/larva (PIAA)). (**C**) Representative Western blot showing increased pS6K1 levels in PIAA-treated recovering larvae. (**D-I’**) Confocal images of [*Tg*(*ins:CFP-NTR*)^*s892*^; *Tg*(*ins:Kaede*)^*jh6*^] larvae at 48 hpa, concurrently treated with EdU and DMSO (**D-D’**), PIAA (**E-E’**), cilostamide (**F-F’**), a combination of PIAA and cilostamide (**G-G’**), a combination of PIAA and rapamycin (**H-H’**), or a combination of cilostamide and rapamycin (**I-I’**), respectively, from 0–48 hpa, stained for pRPS6 (blue). The number of EdU-incorporated (white arrows) and pRPS6-positive (white arrowheads) β-cells was increased in recovering larvae treated with both PIAA and cilostamide (**G**,**G’**) compared to individual compound-treated larvae (**E-E’** and **F-F’**). Rapamycin substantially suppressed the PIAA- and cilostamide-dependent increases in the number of EdU-incorporated and pRPS6-positive β-cells (**H-I’**). (**J**) Quantification of the number (mean ± SD) of total regenerated β-cells (green bars) and regenerated β-cells that incorporated EdU with pRPS6 immunoreactivity (white bars) at 48 hpa (in D-I’; 5.0 ± 1.3 total regenerated β-cells, of which 0.3 ± 0.5 (DMSO), 17.8 ± 2.8, of which 8.4 ± 1.7 (PIAA), 12.7 ± 1.9, of which 5.0 ± 1.5 (cilostamide), 24.7 ± 1.2, of which 13.3 ± 0.6 (PIAA and cilostamide), 6.0 ± 2.0, of which 1.0 ± 1.0 (PIAA and rapamycin), and 5.2 ± 1.1, of which 0.8 ± 0.8 (cilostamide and rapamycin) incorporated EdU with pRPS6 immunoreactivity). (**K**) The percentage (mean ± SD) of regenerated β-cells that incorporated EdU with pRPS6 immunoreactivity at 48 hpa (in D-I’; 6.1 ± 9.5% (DMSO), 47.3 ± 7.5% (PIAA), 50.4 ± 8.8% (cilostamide), 54.2 ± 4.2% (PIAA and cilostamide), 13.9 ± 12.7% (PIAA and rapamycin), and 13.7 ± 13.0% (cilostamide and rapamycin)). Cells in 20 planes of confocal images from 10 individual larvae were counted per condition. ***P < 0.001.

Given the previous studies showing PKA directly phosphorylating mTOR and RAPTOR in white adipose browning via βAR-cAMP-PKA-mTORC1 signaling pathway^42^ and TBK1 and IKKε direct phosphorylating and activating PDE3B *in vitro*^23^, and our replication-pathway inhibitor data (Fig. S5I-J and S5N), we hypothesized that TBK1/IKKε-Is induce the activity of the cAMP-PKA-mTOR for β-cell regeneration via suppression of the TBK1/IKKε-PDE3 signaling axis (Fig. 6A). To test this hypothesis, we first measured the cellular cAMP levels and determined the phosphorylation status of the S6 kinase 1 (S6K1), a downstream target of the mTOR signaling cascade^43^, in the PIAA-treated regenerating larvae. Treatment with PIAA led to pronounced increases in both cAMP levels and S6K1 protein phosphorylation (Figs 6B, C, S6A and S7). Furthermore, a combination of PIAA and PDE3 inhibitor cilostamide enhanced β-cell regeneration with increases in cAMP levels, number of β-cells that were EdU incorporated, and immunoreactive for phosphorylated ribosomal protein S6 (pRPS6), another downstream target of the mTOR signaling cascade^43^, compared to individual compound-treated larvae (Figs 6E-6G’, 6J-6K, S6B, and S8A–E). These effects were suppressed by rapamycin treatment (Figs 6H-6I’, 6J, K, and data not shown). In a converse experiment, we assessed the effects of ectopic expression of *pde3a* on mitogenic potential of TBK1/IKKε-Is using a heat-inducible transgene *Tg*(*hsp*:*pde3a; hsp*:*GFP*)^*gt4*^. Pde3a is the only PDE3 isoform in zebrafish. When *pde3a* expression was induced during recovery period in the presence of PIAA, the proportion of new β-cells, which were EdU incorporated and pRPS6-positive, was decreased compared to PIAA-only-treated larvae (Fig. S8F-K). These data suggest that suppression of TBK1/IKKε bestows an increase in β-cell number by regulating cAMP and mTOR activity through PDE3 in the zebrafish model of type 1 diabetes (Fig. S8L).

### TBK1/IKKε inhibition augments β-cell function and proliferation in mammalian systems

**Figure 7:**
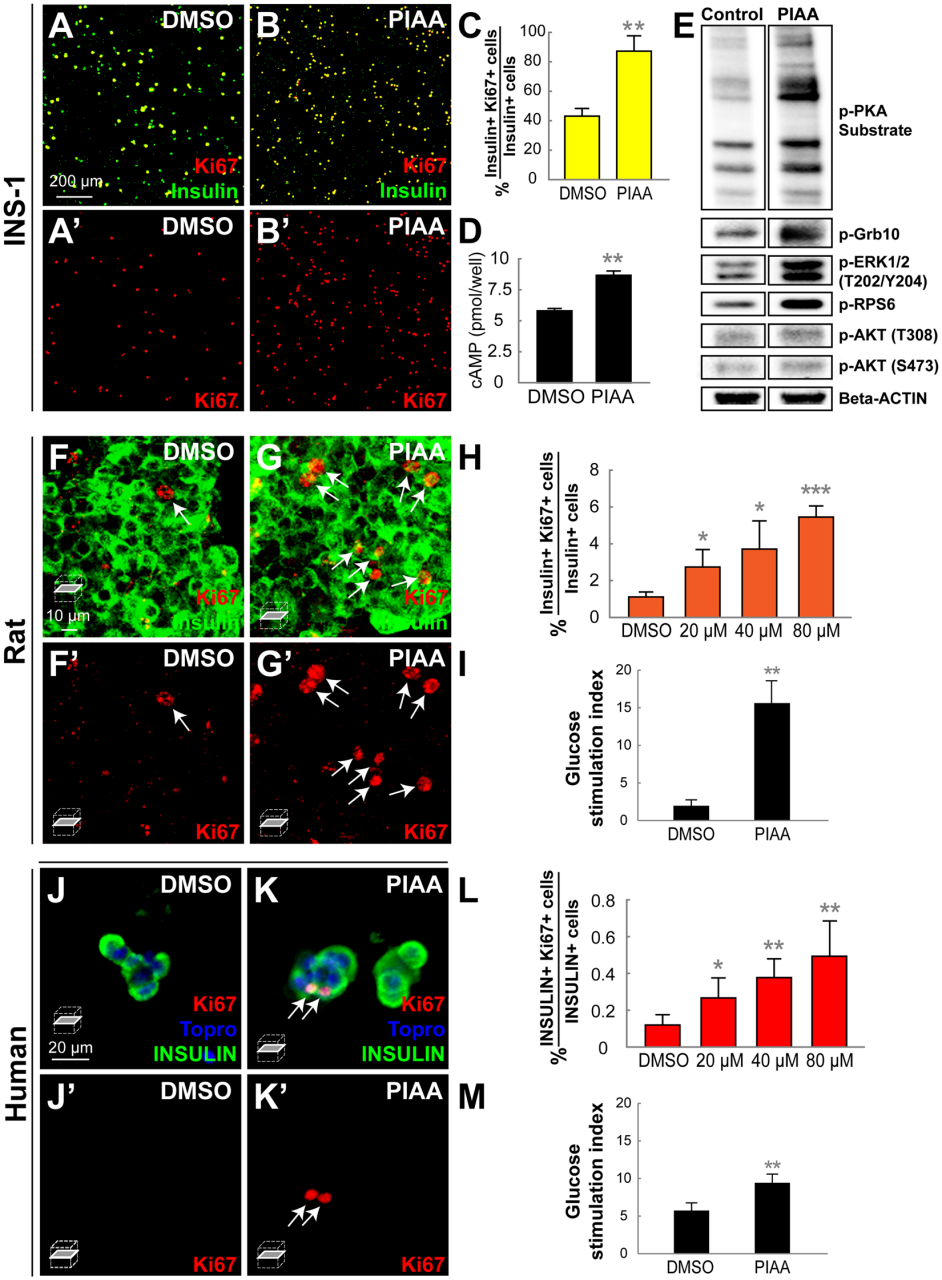
PIAA induces proliferation of rat and human β-cells. (**A-B’**) Confocal images of rat INS-1 β-cells treated with DMSO (**A-A’**) and PIAA (**B-B’**), respectively, stained for Ki67 (red) and Insulin (green). (**C**) The percentage (mean ± SD) of Ki67 and Insulin-double positive cells (in A-B’; 43.1 ± 5.2% (DMSO) and 87.3 ± 10.4% (PIAA)). (**D**) Quantification of cAMP levels (mean ± SD) (5.8 ± 0.2 pmol/well (DMSO) and 8.7 ± 0.4 pmol/well (PIAA)). (**E**) representative Western blot showing increased phosphorylation of PKA substrate and ERK1/2 in PIAA-treated INS-1-derived 832/13 β-cells. Note that PIAA-treatment augmented phosphorylation of mTOR targets RPS6 and Grb10 but did not trigger phosphorylation of AKT^T308^ and AKT^S473^. (**F-G’**) Confocal single-plane images of whole rat islets treated with DMSO (**F-F’**) and PIAA (**G-G’**), respectively, stained for Ki67 (red, white arrows) and Insulin (green). (**H**) The percentage (mean ± SD) of Ki67 and Insulin-double positive cells in whole rat islets increased in a dose-dependent manner with treatment of PIAA (1.1 ± 0.3% (DMSO), 2.7 ± 0.9% (20 μM), 3.7 ± 1.5% (40 μM), and 5.5 ± 0.6% (80 μM)). n = 5 replicates per condition. (**I**) Glucose stimulation indices of rat islets treated with DMSO or PIAA (300 islet equivalents per column, triplicate). (**J-K’**) Confocal single-plane images of human islets treated with DMSO (**J-J’**) and PIAA (**K-K’**), respectively, stained for Ki67 (red, white arrows), Topro (blue), and INSULIN (green). (**L**) The percentage (mean ± SD) of Ki67 and Insulin-double positive cells in human islets increased in a dose-dependent manner with treatment of PIAA (0.1 ± 0.0% (DMSO), 0.3 ± 0.1% (20 μM), 0.4 ± 0.1% (40 μM), and 0.5 ± 0.2% (80 μM)). n = 5 replicates per condition from 3 cadaveric donors. (**M**) Glucose stimulation indices of human islets treated with DMSO or PIAA (300 islet equivalents per column, triplicate). *P < 0.05; **P < 0.01; ***P < 0.001.

To determine whether the effects of TBK1/IKKε suppression on β-cells are conserved across species, we first performed glucose-stimulated insulin secretion (GSIS) assay in primary rat and human islets as elevation of cAMP levels has shown to lead to enhanced β-cell replication, survival, and insulin secretion^10,28,29^. PIAA treatment significantly increased glucose stimulation indices in rat and human islets (Fig. 7I, M). Next, we investigated the mitogenic effects of TBK1/IKKε inhibition by analyzing the ability of PIAA to increase β-cell proliferation in INS-1 cells^60^. Treating INS-1 cells with PIAA resulted in increased percentage of proliferating Insulin-positive cells (co-expressed Ki-67) (Fig. 7A–C and Supplementary Table 1) and levels of cAMP (Fig. 7D). Furthermore, PIAA augmented phosphorylation of PKA substrate and S6K1, RPS6, and Grb10 proteins, which are established targets of mTOR^61^, as well as ERK1/2, whereas it triggered minimal phosphorylation of Akt (Figs 7E, S9A, B, and S10) in INS-1 and INS-1-derived 832/13 cells^62^. These data suggest that suppression of TBK1/IKKε promotes replication of β-cells via activation of the cAMP-mTOR signaling axis in mammalian systems. PIAA also increased β-cell proliferation in both dispersed and whole rat islets in a dose-dependent manner (Fig. 7F–H, Supplementary Table 2, and data not shown), consistent with its mitogenic effect on β-cell formation even in the absence of injury in zebrafish (Fig. S11A–D). Importantly, treatment of PIAA on primary human β-cells using islets obtained from 3 cadaveric organ donors caused a notable, dose-dependent induction of β-cell proliferation (Fig. 7J–L and Supplementary Table 3).

**Figure 8:**
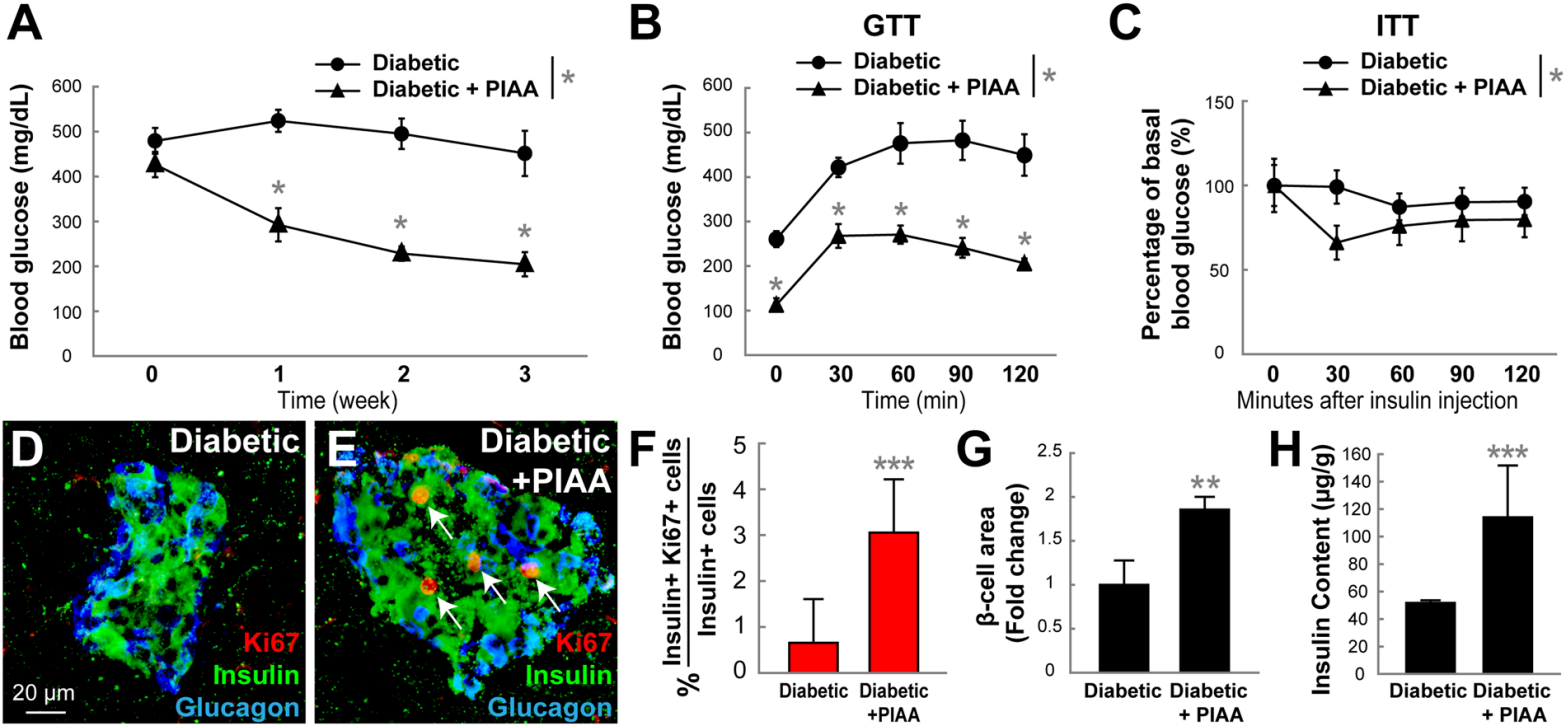
PIAA improves glucose control and β-cell mass in the STZ-induced diabetic mouse model. (**A**–**H**) STZ-induced diabetic mice were treated with vehicle or PIAA for 2–3 weeks after reaching > 300 mg/dL fed glucose values (n = 6–8 mice per group). (**A**) PIAA caused reduction of hyperglycemia (non-fasting glucose measurement) relative to vehicle-treated animals. PIAA-treated animals showed improved (**B**) glucose and (**C**) insulin tolerance. Confocal images of diabetic pancreata treated with (**D**) vehicle and (**E**) PIAA, respectively, stained for Ki67 (red, white arrows), Insulin (green), and Glucagon (blue). (**F**) The percentage (mean ± SD) of Ki67 and Insulin-double positive cells in diabetic islets increased with PIAA treatment (0.7 ± 1.0% (vehicle) and 3.1 ± 1.2% (PIAA)). Quantification (mean ± SD) of (**G**) β-cell area (fold change, 1.0 ± 0.3 (vehicle) and 1.9 ± 0.1 (PIAA)) and (**H**) insulin content (51.8 ± 1.9 μg/g (vehicle) and 114.0 ± 37.7 μg/g (PIAA)) in diabetic pancreata treated with vehicle or PIAA. *P < 0.05; **P < 0.01; ***P < 0.001.

We further investigated whether PIAA could increase β-cell regeneration in the streptozotocin (STZ)-induced mouse model of type 1 diabetes. PIAA administration started causing a substantial reduction of non-fasting blood glucose levels after 4–5 days of intraperitoneal injection (Fig. 8A). Significant improvement in glucose and insulin tolerance was observed compared with vehicle treatment (Fig. 8B, C). Morphometric analysis of pancreas sections showed that the β-cells, not α-cells, in PIAA-treated mice were more likely to be Ki67^+^, indicating that they were proliferating at a higher rate (Fig. 8D–F and Supplementary Table 4). Moreover, β-cell area and insulin content were increased in PIAA-treated compared with vehicle-treated diabetic mice (Fig. 8G, H). There was no difference in the weight of the mice based on treatment, neither at the start nor at the end of the experiments, indicating that the mice were not generally affected by PIAA treatment (data not shown). Taken together, these data suggest that inhibition of TBK1/IKKε leads to improvement of β-cell function and induction of β-cell replication across multiple species including primary human β-cells and diabetic mice.

## Discussion

In this study, we identified TBK1/IKKε-Is as selective enhancers of β-cell regeneration in a transgenic zebrafish model of type 1 diabetes. We further demonstrated that inhibition of TBK1/IKKε promotes amplification of β-cells in mammalian systems including primary rat and human islets as well as STZ-induced diabetic mice. The proliferative effects of TBK1/IKKε-Is are likely to be mediated by the cAMP-PKA-mTOR signaling axis via PDE3, indicating that TBK1/IKKε play a previously unappreciated role in modulating β-cell mass.

Utilizing small molecule inducers of β-cell proliferation is one of the most tangible methods to restore functional β-cell mass. We have pinpointed that inhibition of TBK1/IKKε promotes β-cell proliferation in multiple species including primary human β-cells. Of essential importance is that suppression of TBK1/IKKε using amlexanox and PIAA, which exhibited the highest potency among tested, can increase β-cell proliferation selectively without inducing a general increase in proliferation of other cell types/tissues. Furthermore, a longer treatment of β-cell ablated zebrafish with these small molecules did not lead to over-proliferation of β-cells once normoglycemia was approached. Since oncogenicity can arise as a result of modulating mitogenic or regenerative pathways^4^, specific TBK1/IKKε-Is’ selectivity to β-cells and ability to increase β-cell proliferation primarily during the most active period of β-cell regeneration present valid strategies for expanding β-cell mass. Furthermore, PIAA exhibited substantial efficiency in β-cell regeneration with minimum toxicity. Biologically less characterized azabenzimidazole (AZ) derivatives 5c and 5e have IC50 of 0.032 μM and 0.102 μM (AZ-5c) as well as 0.038 μM and 0.204 μM (AZ-5e) against TBK1 and IKKε, respectively^51^, compared to that of 0.4 μM and 1.07 μM (PIAA). Another AZ derivative AZ13102909 has an IC50 of 0.005 μM against TBK1, promoting apoptosis in melanoma cells^63^. It is noteworthy that AZ-5c and AZ-5e demonstrated significant toxicity when testing in the β-cell ablated zebrafish at nM range with less efficiency of β-cell regeneration than PIAA (data not shown). Diarylamide WS6, which was previously suggested to target IKKε with increasing β-cell proliferation potency in primary human islet culture^9,11^, increased β-cell regeneration much less efficiently than PIAA in the zebrafish model of type 1 diabetes (data not shown). Our results support a separate study showing that WS6 had modest effects on human β-cell proliferation^12^. Therefore, further design and validation of new molecular structures with potent TBK1 and/or IKKε inhibition activities and minimal toxicity using the PIAA as a scaffold will allow us to identify legitimate strategies for developing human β-cell-specific proliferogens.

Previous studies have suggested that modulation of cAMP levels via GPCR in β-cells is essential for β-cell replication, survival, and insulin secretion^10,28,29^. Our results provide compelling evidence that inhibition of TBK1/IKKε enhances selective β-cell proliferation by increasing cAMP levels via PDE3. Time-course analysis in zebrafish treating PDE3 inhibitor cilostamide, PIAA, and a combination of PIAA and cilostamide demonstrated the correlation between the levels of cAMP and expansion of β-cell mass: the most dynamic period of β-cell regeneration corresponds to high levels of cAMP, whereas the period reaching to normoglycemia without over-proliferation of β-cells links to low levels of cAMP. Moreover, PIAA treatment of INS-1 rat β-cells resulted in increased percentage of proliferating Insulin-positive cells (co-expressed Ki-67) with concurrent augmentation of cAMP levels. It is likely that PKA stimulation is responsible for linking PIAA-enhanced cAMP levels and subsequent β-cell proliferation. In INS-1 and INS-1-derived 832/13 cells, PIAA increased phosphorylation of PKA substrate and ERK1/2, which was shown to be driven β-cell proliferation via cAMP-PKA signaling cascade upon GIP/GIPR stimulation^35^. Phosphorylation of mTOR targets including S6K1, RPS6, and Grb10, was also greatly augmented with PIAA treatment. Both mammalian and zebrafish mTOR proteins contain 3 conserved PKA target RR/KXS motifs and RAPTOR proteins have 1 RRXS motif (JX and CHS, unpublished observation) that were directly phosphorylated by PKA upon treatment with βAR agonists *in vitro*^42^. PIAA did not trigger phosphorylation of Akt^T308^ and Akt^S473^, suggesting that the effect of TBK1/IKKε repression is at least in part through a cAMP-PKA-mTOR signaling, not via a well-established Akt-mTOR signaling. The suggested suppressive effect of PI3K-Akt signaling in β-cell regeneration in our studies is consistent with a report showing the inhibitory role of PI3K signaling in β-cell formation in zebrafish^64^ and may act through PDE3. Akt has been demonstrated to phosphorylate PDE3B *in vitro*^23,65^ and PI3K has shown to directly activate Akt1 and stimulate Akt2 in a PDPK1-dependent manner^66^. Consistently, treatment of cilostamide with PI3K and Akt inhibitors led to increase in β-cell regeneration in zebrafish (data not shown). In this regard, PDPK1 triggering activation of both Akt and S6K1^67^ may be the reason for minimal increase in β-cell number with PDPK1 inhibition. While α_2_-adrenergic receptor antagonist mirtazapine and several PDE inhibitors including a PDE3 inhibitor cilostamide have displayed their potency to stimulate β-cell replication in a cAMP-dependent manner^10^, enhanced insulin secretion was observed in *Pde3b* knockout (KO) mice^68^. However, *Pde3b* KO mice fail to suppress hepatic glucose production and display insulin resistance with a number of cAMP-signal transduction components being altered in *Pde3b*-deficient livers^68^. Contrarily, genetic deletion of IKKε and pharmacological inhibition of TBK1/IKKε led to improved insulin sensitivity through the inhibition of hepatic glucose production with decrease in PDE3B activity and increase in cAMP levels in adipocytes, not in livers, in obese mice^50,69^. Thus, despite the ameliorated insulin tolerance in PIAA-treated STZ diabetic mice may be a secondary effect of hyperglycemic reversal by augmenting β-cell mass, our findings of modulation of PDE3 activity and cAMP levels via suppression of TBK1/IKKε is likely to enable us to pinpoint bona fide therapeutic approaches that increase the number of functionally adequate β-cells with direct or indirect improvement of insulin sensitivity.

In progression of T1 and T2DM, decreasing β-cell mass by cytokine- and/or glucolipotoxicity-induced apoptosis is a common feature. Thus, prevention of β-cell loss can be an alternative approach for increasing β-cell mass in diabetes. Considering our studies demonstrating that repression of TBK1/IKKε increases cAMP levels and the previous studies showing that anti-apoptotic gene *bcl2* is induced by the cAMP-PKA-cAMP response element binding protein (CREB) signaling axis^38^, it is plausible to speculate that suppression of TBK1/IKKε can preserve β-cell mass. Intriguingly, adipose-specific genetic ablation of TBK1 attenuates diet-induced obesity with exaggeration in glucose intolerance/insulin resistance, while genetic deletion of IKKε increases energy expenditure with improvement in insulin sensitivity on a high fat diet^70^. Thus, a careful dissection and elucidation of TBK1- and/or IKKε-controlled signaling networks will shed light on modulating β-cell survival with concomitant increase in functional β-cell mass, opening up new avenues of therapies for mitigating diabetes.

## Experimental Procedures

### Zebrafish strains

Adult fish and embryos/larvae were raised and maintained under standard laboratory conditions^71^. We used the following published transgenic lines: *Tg*(*ins*:*CFP-NTR*)^*s892*47^ and *Tg*(*ins:Kaede*)^*jh6*48^. Zebrafish studies conducted and protocols used were approved by the Institutional Animal Care and Use Committees of Mayo Clinic and Georgia Institute of Technology, and were in accordance with National Institutes of Health guidelines.

### β-cell ablation, chemical treatment, and photoconversion

To ablate β-cells, [*Tg*(*ins*:*CFP-NTR*)^*s892*^; *Tg*(*ins:Kaede*)^*jh6*^] larvae were treated with freshly prepared 5 mM metronidazole (MTZ) (Sigma) from 3 dpf to 4 dpf in the dark, followed by washing out the MTZ, and subsequent 48 hours recovery in the presence of DMSO, individual, or combination of the chemical compounds. The names and the concentrations of the chemical compounds are described in the Supplemental Experimental Procedures. To demonstrate that all β-cells are ablated by MTZ treatment, *Tg*(*ins:*Kaede) ^*jh6*^-expressing β-cells were converted from green to red by exposing them to UV light at 3 dpf immediately after MTZ treatment.

### Chemical screening

To perform a chemical screen for compounds enhancing β-cell regeneration, we tested 75 compounds from the Stem Cell Signaling Compound Library (Selleckchem). Further details are described in the Supplemental Experimental Procedures.

### Immunohistochemistry

Immunohistochemistry on whole-mount zebrafish larvae and 5-ethynyl-2′-deoxyuridine (EdU) analysis were performed as previously described^72,73^. For mammalian islet culture, after chemical treatment, islets were washed with PBS and fixed in 4% paraformaldehyde (PFA) for 1 hour. For mouse studies, 8 μm thick sections were obtained by using a cryostat microtome (CryoStar NX70 Cryostat). See Supplemental Experimental Procedures for additional information.

### Glucose and cAMP measurements

Glucose measurements were performed 3 times on 10 zebrafish larvae per condition using a fluorescence-based enzymatic detection kit (Biovision Inc.). cAMP content of whole-zebrafish larvae and rat INS-1 cells was analyzed using commercial ELISA kits (Enzo Life Sciences, Inc.). Further details are described in the Supplemental Experimental Procedures.

### Mammalian *ex vivo* islet culture and mice experiments

Male Lewis rat pancreatic donors were purchased from Charles River (Wilmington, MA).

Human islets from healthy donors were purchased from Prodo Laboratories (Irvine, CA). 6–8-wk old C57BL/6 male mice (Jackson Laboratory) were used. Studies conducted and protocols used were approved by the Institutional Animal Care and Use Committee of Mayo Clinic and Georgia Institute of Technology and were in accordance with National Institutes of Health guidelines. See Supplemental Experimental Procedures for additional information.

### Statistical analysis

All statistical analyses were performed using GraphPad Prism (version 7). *P*-values less than 0.05 were considered statistically significant.

## Electronic supplementary material

Supplementary Information
