# Publisher Correction: Inhibition of TBK1/IKKε Promotes Regeneration of Pancreatic β-cells

**DOI:** 10.1038/s41598-019-54301-z

**Published:** 2019-12-03

**Authors:** Jin Xu, Yun-Fang Jia, Subhasish Tapadar, Jessica D. Weaver, Idris O. Raji, Deeti J. Pithadia, Naureen Javeed, Andrés J. García, Doo-Sup Choi, Aleksey V. Matveyenko, Adegboyega K. Oyelere, Chong Hyun Shin

**Affiliations:** 1 School of Biological Sciences and the Parker H. Petit Institute for Bioengineering and Bioscience, Georgia Institute of Technology, Atlanta, GA 30332 USA; 2 Department of Molecular Pharmacology and Experimental Therapeutics, Mayo Clinic, Rochester, MN 55905 USA; 3 School of Chemistry and Biochemistry and the Parker H. Petit Institute for Bioengineering and Bioscience, Georgia Institute of Technology, Atlanta, GA 30332 USA; 4 Woodruff School of Mechanical Engineering and the Parker H. Petit Institute for Bioengineering and Bioscience, Georgia Institute of Technology, Atlanta, GA 30332 USA; 5 Department of Physiology and Biomedical Engineering, Mayo Clinic, Rochester, MN 55905 USA; 6 Present Address: Division of Biology and Biological Engineering, California Institute of Technology, Pasadena, CA 91125 USA

Correction to: *Scientific Reports* 10.1038/s41598-018-33875-0, published online 22 October 2018

In the original HTML version of this Article, Yun-Fang Jia was incorrectly affiliated with ‘Division of Biology and Biological Engineering, California Institute of Technology, Pasadena, CA, 91125, USA’.

This error has now been corrected in the HTML version of the Article, the PDF version was correct at the time of publication.

