# Supplementary material for: Inhibition of TBK1/IKKε Promotes Regeneration of Pancreatic β-cells
Source: Sci Rep. 2018 Oct 22;8:15587. doi: 10.1038/s41598-018-33875-0 (PMC6197228; doi:10.1038/s41598-018-33875-0)
Supplement: Supplementary file 1 — Supplementary Information [file 41598_2018_33875_MOESM1_ESM.pdf]

Inhibition of TBK1/IKKε Promotes Regeneration of Pancreatic β-cells

Jin Xu, Yun-Fang Jia, Subhasish Tapadar, Jessica D. Weaver, Idris O. Raji, Deeti J. Pithadia, Naureen Javeed, Andrés J. García, Doo-Sup Choi, Aleksey V. Matveyenko, Adegboyega K. Oyelere, Chong Hyun Shin

Supplementary Information

Supplemental Data

Figure S1. Synthesis of PIAA, PIAA analogs, and *iso*-PIAA. (A) Scheme for synthesis of PIAA and its analogs. Reagents & Conditions: (a) methyl acrylate, $Pd(OAc)_2$, P(*o*-tolyl)$_3$, Et_3N, CH_3CN, 90 °C, 6 h. (b) LiOH, THF-MeOH-H_2O (3:1:1), room temp., 10 h. (c) acrylamide, $Pd(OAc)_2$, P(*o*-tolyl)$_3$, Et_3N, CH_3CN, 90 °C, 6 h. (B) Scheme for synthesis of *iso*-PIAA. Reagents & Conditions: (a) $NaNO_2$, AcOH, H_2O, 0 °C, 1 h, then NaN3, room temp., 2 h; (b) $FeBr_2$ (5 mol%), 4Å molecular sieves (100 mol%), CH_2Cl_2, 40 °C, 16 h. (c) methyl acrylate, $Pd(OAc)_2$, P(*o*-tolyl)$_3$, Et_3N, CH_3CN, 90 °C, 6 h. (d) LiOH, THF-MeOH-H_2O (3:1:1), room temp., 3 h.

Figure S2. Full western blot scans for Fig. 2J. The full scans for each blot are shown right.

Figure S3. TBK1/IKKε-Is augment the number of the newly formed β-cells. (A) Schematic diagram for assessment of ablation efficiency of β-cells. (B) Quantification of the number (mean±SD) of newly regenerated (Green$^+$) β-cells at 48 hpa (4.4±1.6 β-cells were green-only-positive in DMSO-treated larvae, while 9.3±2.2 (BX795), 11.1±4.4 (amlexanox), and 18.1±3.6 (PIAA) β-cells expressed as green-only in TBK1/IKKε-I-treated recovering larvae). Cells in 20 planes of confocal images from 10 individual larvae were counted per condition. ***, $P < 0.001$.

Figure S4. TBK1/IKKε inhibitors do not increase proliferation of liver cells nor lead to an overshoot in β-cell number. (A-C) Confocal single-plane images of [*Tg(ins:CFP-NTR)*^{s892};

Tg(ins:Kaede)^{jh6}] larvae at 48 hpa, concurrently treated with EdU and DMSO (A), amlexanox (B), or PIAA (C), respectively, from 0-48 hpa, stained for Prox1 (blue). The number of Prox1-positive cells in the liver that incorporated EdU did not significantly increase in TBK1/IKKε-I-treated recovering larvae (B and C) compared to DMSO-treated larvae (A). (D) The percentage (mean±SD) of Prox1-positive liver cells that incorporated EdU at 48 hpa (in A-C; 31.6±5.6% (DMSO), 30.1±5.8% (amlexanox), and 33.5±10.5% (PIAA)). Cells in 10 planes of confocal images from 5 individual larvae were counted per condition. (E-G) Bright-field images combined with fluorescent images showing the overall morphology of embryos and [*Tg(ins*:CFP-NTR*)^{s892}*; *Tg(ins*:Kaede*)^{jh6}*] expression (green) in larvae at 96 hpa treated with DMSO (E), amlexanox (F), and PIAA (G), respectively. While TBK1/IKKε-Is expanded [*Tg(ins*:CFP-NTR*)^{s892}*; *Tg(ins*:Kaede*)^{jh6}*]-expressing cell population (white squares and insets) during regeneration (F and G) compared to DMSO (E), a longer treatment (0-96 hpa) did not result in overproliferation of β-cells. (H-J) Confocal single-plane images of [*Tg(ins:CFP-NTR)^{s892}*; *Tg(ins:Kaede)^{jh6}*] larvae at 96 hpa (in E-G), stained with Topro (blue). White arrows indicate β-cells located in secondary islets.

Figure S5. TBK1/IKKε suppression promotes β-cell regeneration via PKA and mTOR signaling axis. (A-L) Bright-field images combined with fluorescent images showing the overall morphology and [*Tg(ins*:CFP-NTR*)^{s892}*; *Tg(ins*:Kaede*)^{jh6}*] expression (green) of larvae at 48 hpa treated with DMSO (A), calcineurin inhibitor FK506 (B), mTOR inhibitor rapamycin (C), PKA inhibitor PKI-(6-22)-amide (D), PI3K inhibitor LY294002 (E), Akt inhibitor MK2206 (F), PIAA (G), and combinations of each inhibitor with PIAA (H-L), respectively. Treatment of rapamycin and PKI-(6-22)-amide suppressed PIAA-mediated β-cell regeneration (G vs. I and J). The

individual LY294002 and MK2206 caused increase in the [*Tg(ins*:CFP-NTR*)*^{s892}; *Tg(ins*:Kaede*)*^{jh6}]-expressing cell population during regeneration (E and F). The combinations of LY294002 or MK2206, especially MK2206, with PIAA resulted in significant increase in β-cell regeneration (K and L) compared to individual treatments (E and F). The insets display magnified views of the pancreatic islets (outlined by the dashed squares). (M-N) Quantification of the number (mean±SD) of total regenerated β-cells at 48 hpa (in A-L; 4.7±1.2 (DMSO), 5.5±1.7 (FK506), 4.2±1.3 (rapamycin), 5.4±1.7 (PKI-(6-22)-amide), 8.7±1.7 (LY294002), 14.8±2.7 (MK2206), 17.9±2.4 (PIAA), 16.7±2.3 (PIAA with FK506), 7.0±2.1 (PIAA with rapamycin), 9.6±2.8 (PIAA with PKI-(6-22)-amide), 21.3±4.3 (PIAA with LY294002), 31.9±5.1 (PIAA with MK2206)). Cells in 20 planes of confocal images from 20 individual larvae were counted per condition. **, $P < 0.01$; ***, $P < 0.001$. (O) A model depicting the role of PKA and mTOR signaling pathways in mediating the β-cell regeneration response to TBK1/IKKε inhibition. The site of action of PIAA is shown in red, while those of LY294002 and MK2206 in blue.

Figure S6. Dynamics of cAMP levels correlate with expansion of β-cell mass. (A) Quantification of cAMP levels (mean±SD) during β-cell regeneration in DMSO- and PIAA-treated recovering larvae. Unlike 1 and 4 days-post-ablation (dpa), cAMP levels were significantly higher in PIAA-treated recovering larvae than in DMSO-treated larvae at 2 dpa (1 dpa: 0.5±0.1pmol/larva (DMSO) and 0.6±0.0 pmol/larva (PIAA); 2 dpa: 0.5±0.1pmol/larva (DMSO) and 0.9±0.0 pmol/larva (PIAA); 4 dpa: 0.4±0.0 pmol/larva (DMSO) and 0.4±0.2 pmol/larva (PIAA)). (B) Quantification of cAMP levels in DMSO-, cilostamide-, PIAA-, and a combination of cilostamide and PIAA-treated recovering larvae at 2 dpa. A combination of

cilostamide and PIAA augmented cAMP levels compared to individual compound-treated larvae (0.5±0.1 pmol/larva (DMSO), 0.8±0.1 pmol/larva (cilostamide), 0.9±0.0 pmol/larva (PIAA), and 1.4±0.2 pmol/larva (cilostamide and PIAA)). **, $P < 0.01$; ***, $P < 0.001$. 10 larvae were pooled per condition and data point.

Figure S7. Full western blot scans for Fig. 6C. The full scans for each blot are shown right.

Figure S8. Suppression of TBK1/IKKε bestows an mTOR-mediated increase in β-cell number via PDE3. (A-D) Bright-field images combined with fluorescent images showing the overall morphology and [*Tg(ins*:CFP-NTR*)*^{s892}; *Tg(ins*:Kaede*)*^{jh6}] expression (green) of larvae at 48 hpa treated with DMSO (A), cilostamide (B), PIAA (C), and PIAA with cilostamide (D), respectively. The combination of cilostamide with PIAA resulted in increase in the [*Tg(ins*:CFP-NTR*)*^{s892}; *Tg(ins*:Kaede*)*^{jh6}]-expressing cell population during regeneration (D) compared to individual treatment (B and C). The insets display magnified views of the pancreatic islets (outlined by the dashed squares). (E) Quantification of the number (mean±SD) of total regenerated β-cells at 48 hpa (in A-D; 4.5±1.0 (DMSO), 11.6±2.2 (cilostamide), 18.2±2.6 (PIAA), and 24.6±3.6 (cilostamide with PIAA). Cells in 20 planes of confocal images from 20 individual larvae were counted per condition. ***, $P < 0.001$. (F-I') Confocal images of EdU-administered [*Tg(ins:CFP-NTR)*^{s892}; *Tg(ins:Kaede)*^{jh6}] larvae at 48 hpa, treated with DMSO (F and F') and PIAA (H and H'), respectively, or induced *pde3a* expression upon β-cell ablation (G and G') and *pde3a* expression upon β-cell ablation in the presence of PIAA (I and I'), respectively, from 0-48 hpa, stained for pRPS6 (blue). The number of EdU-incorporated (white arrows) and pRPS6-positive (white arrowheads) β-cells was increased in recovering larvae

treated with PIAA (H and H') compared to DMSO-treated larvae (F and F'). When *pde3a* expression was induced upon β-cell ablation, PIAA-dependent increases in the number of EdU-incorporated and pRPS6-positive β-cells were suppressed (I and I'). (J) Quantification of the number (mean±SD) of total regenerated β-cells (green dots) and regenerated β-cells that incorporated EdU with pRPS6 immunoreactivity (white dots) at 48 hpa (in F-I'; 4.8±1.2 total regenerated β-cells, of which 0.5±0.5 (DMSO), 4.0±1.0, of which 0.4±0.5 (*pde3a* overexpressing), 17.8±2.8, of which 8.0±1.9 (PIAA), and 8.0±2.8, of which 1.4±1.1 (*pde3a* overexpressing in the presence of PIAA) incorporated EdU with pRPS6 immunoreactivity). (K) The percentage (mean±SD) of regenerated β-cells that incorporated EdU with pRPS6 immunoreactivity at 48 hpa (in F-I'; 8.9±9.8% (DMSO), 8.0±11.0% (*pde3a* overexpressing), 44.8±7.6% (PIAA), and 15.3±9.9% (*pde3a* overexpressing in the presence of PIAA)). Cells in 20 planes of confocal images from 10 individual larvae were counted per condition. ***, $P < 0.001$. (L) A model depicting the cAMP-PKA-mTOR signaling cascade via PDE3 that mediates β-cell regeneration in response to TBK1/IKKε inhibition. The site of action of PIAA is shown in red, while those of LY294002 and MK2206 in blue, and cilostamide in purple.

Figure S9. PIAA increases mTOR activity. Representative Western blot showing (A) increased pS6K1 levels with PIAA-treatment in rat INS-1 cells. The full scans for each blot are shown in (B).

Figure S10. Full western blot scans for Fig. 7E. The full scans for each blot are shown right.

Figure S11. PIAA increases β-cell proliferation during normal development in zebrafish. (A-B) Confocal images of [*Tg(ins:CFP-NTR)*^{s892}; *Tg(ins:Kaede)*^{jh6}] larvae at 6 dpf, concurrently treated EdU and DMSO (A) or PIAA (B), respectively, from 4-6 dpf. A small but significant increase in EdU incorporation (white arrows) was observed in PIAA-treated larvae (B) compared to DMSO-treated larvae (A) during normal development. (C) Quantification of the number (mean±SD) of total β-cells (green dots) and β-cells that incorporated EdU (yellow dots) (in A-B; 38.0±3.2 total β-cells, of which 5.8±1.0 (DMSO) and 45.3±9.3, of which 11.0±3.6 (PIAA) incorporated EdU). (D) The percentage (mean±SD) of β-cells that incorporated EdU (in A-B; 15.2±2.7% (DMSO) and 23.8±3.4% (PIAA)). Cells in 20 planes of confocal images from 5 individual larvae were counted per condition. *, $P < 0.05$.

Supplemental Experimental Procedures

Generation of *pde3a*-overexpressing zebrafish

To generate the *Tg(hsp:pde3a; hsp:GFP)*^{gt4}, *pde3a* coding sequence was amplified (forward: 5'-GACGAATTCGTGACCGATGGCACTGGAAT-3', reverse: 5'-GACCTCGAGTCATTCTGGATCTTTCTGGTCTTCG-3') and cloned into a vector containing a multimerized minimal heat shock promoter, which drives *gfp* and *pde3a* transcription bi-directionally in response to a heat shock[1]. Tol2-mediated transgenesis was achieved as described[2].

Chemical Screening

To perform chemical screens, we first aliquoted each chemical compound into two separate wells of a 96-well glass bottom microwell plate (Matrical Bioscience) to a final concentration of 50 μM. Control larvae were treated with equal concentrations of DMSO in egg water. MTZ-treated [*Tg*(*ins*:*CFP-NTR*)^{s892}; *Tg(ins:Kaede)*^{jh6}] larvae were used. Five ablated larvae were pipetted into each well. At 48 hpa, the number and intensity of Kaede-positive cells within each larval pancreas were determined using a Leica M205 FA fluorescence microscope. Five compounds that changed the number and/or intensity of Kaede-positive cells within the pancreas in at least two larvae in both wells were retested at a range of concentrations from 10-100 μM. Two of the compounds, LY411575, a γ-secretase inhibitor, and BX795, a dual suppressor of TBK1/IKKε and PDPK1, showed higher efficiency in β-cell regeneration at 10 μM and 50 μM respectively, on retesting. BX795 was further evaluated by examining the DMSO- and compound-treated regenerating β-cells in [*Tg*(*ins*:*CFP-NTR*)^{s892}; *Tg(ins:Kaede)*^{jh6}] larvae with a Zeiss LSM 700-405 confocal microscope.

Chemical treatment

The following compounds and the concentrations are used in zebrafish: 100 μM amlexanox, 50 μM BX795, 20 μM (E)-3-(3-phenylbenzo[c]isoxazol-5-yl)acrylic acid (PIAA), 25 μM cilostamide, 1 μM rapamycin, 50 μM BX912, 12.5 μM LY294002 (except when combined with PIAA and cilostamide, 1.25-6.25 μM), 10 μM MK2206, 10 μM PKI-(6-22)-amide, 10 μM FK506, 1 μM azabenzimidazole derivative 5c and 5e (kindly provided by Dr. Albert S. Baldwin, Lineberger Comprehensive Cancer Center, University of North Carolina at Chapel Hill), and 20 μM PIAA-1, PIAA-2, PIAA-3, and PIAA-4.

Immunohistochemistry

Immunohistochemistry on whole-mount zebrafish larvae was performed using the following antibodies: mouse anti-Glucagon (1:100; Sigma), chicken anti-GFP (1:1000; Aves Labs), rabbit anti-Somatostatin (1:100; MP Biomedicals), rabbit anti-Prox1 (1:100; GeneTex), rabbit anti-phospho-S6 ribosomal protein (Ser240/244) (1:100; Cell Signaling), and fluorescently conjugated Alexa antibodies (1:200; Molecular Probes). Nuclei were visualized with TOPRO (1:10000; Molecular Probes). To assess cell proliferation, zebrafish larvae were incubated with 7μM 5-ethynyl-2'-deoxyuridine (EdU) and DMSO at 1.7% final concentration for the indicated time period. EdU incorporation was revealed for 30 minutes using Click-iT EdU Imaging Kit (Invitrogen), prior to primary antibody staining. Larvae were mounted in Vectashield (Vector Laboratories) and imaged on a Zeiss LSM 700-405 confocal microscope. Rabbit anti-Ki67 (1:100; Abcam) and guinea pig anti-Insulin (1:100; Sigma) antibodies were additionally used for immunohistochemistry on rat INS-1 cells, mammalian islets in culture, and mice pancreata. Nuclei were visualized with TOPRO (1:2000; Molecular Probes). Rat INS-1 cells and islet samples were directly imaged in plates on a Zeiss LSM 700-405 confocal microscope. Mice pancreata were dissected, fixed in 4% PFA, treated with a 30% sucrose solution, then embedded in Tissue-Tek OCT compound (Sakura Finetek). 8 μm-thick sections were obtained by using a cryostat microtome (CryoStar NX70 Cryostat), stained with antibodies, mounted in Vectashield (Vector Laboratories), and imaged on a Zeiss LSM 700-405 confocal microscope. Two 8 μm-thick pancreas sections (separated by 400 μm) per mouse were used to assess β-cell area. Images were taken after Insulin staining using a Zeiss LSM 700-405 confocal microscope, and islet size/area and the total areas of the sections were determined using the ZEN software (Zeiss). The

percentage of β-cell area in each section was calculated by dividing the area of all insulin-positive cells in one section by the total area of this section.

Western blot analysis

Zebrafish trunks containing the digestive organs from 100 larvae (per experiment) were dissected and extracted in lysis buffer (50 mM Tris–HCl, pH 7.4, 150 mM NaCl, 1 mM $MgCl_2$, 0.5% IGEPAL, freshly added Protease Inhibitor Cocktail and PhosSTOP tablet from Sigma). The rat INS-1 and its derivative INS-1 832/13 cells were cultured in RPMI 1640 (Gibco) supplemented with 10% FBS, 1% Penicillin/Streptomycin, 10 mM HEPES, 1 mM sodium pyruvate, and 50 μM β-mercaptoethanol and used for experiments between passages 6-10. Cells were seeded in 6-well plates at $5x10^5$ cells per well and starved for 18 hours in serum-free or 0.1% endotoxin-free BSA containing RPMI (2.5 mM glucose) prior to exposure to (E)-3-(3-phenylbenzo[c]isoxazol-5-yl)acrylic acid (PIAA) for 30 minutes to 1 hour. After treatment, cells were lysed in lysis buffer mentioned above. Proteins were detected with the following antibodies (Cell Signaling): rabbit anti-phospho-p70 S6 kinase (Thr389) (1:500; product #9205), rabbit anti-p70 S6 kinase (1:1000; product #2708), rabbit anti-p70 S6 kinase (1:1000; product #9202), rabbit anti-IRF-3 (1:1000; product #4302), rabbit anti-phospho-IRF-3 (Ser396) (1:1000; product #29047), rabbit anti-Akt (pan) (1:1000; product #4691), rabbit anti-phospho-Akt (Thr308) (1:1000; product #13038), rabbit anti-phospho-Akt (Ser473) (1:1000; product #9271), rabbit anti-p44/42 MAPK (Erk1/2) (1:1000; product #9102), rabbit anti-phospho-p44/42 MAPK (Erk1/2) (Thr202/Tyr204) (1:1000; product #4370), rabbit anti-phospho-Grb10 (Ser476) (1:1000; product #11817), rabbit anti-phospho-S6 ribosomal protein (Ser240/244) (1:1000; product #5364), mouse anti-S6 ribosomal

protein (1:1000; product #2317), rabbit anti-phospho-PKA substrate (RRXS*/T*) (1:1000; product #9624), and HRP-conjugated secondary antibodies (1:2500).

Glucose and cAMP measurements

For free glucose measurements, the zebrafish larvae were collected in 2 mL microcentrifuge tubes and frozen on crushed dry ice after removal of excess egg water. Upon thawing, 200 μL of PBS were added and the larvae were homogenized using a hand-held mechanical homogenizer. To measure glucose in larval extracts, 15 μL of supernatant was used and mixed for each reaction, and incubated for 30 minutes at 37°C in the dark. Fluorescence (excitation, 535 nm; emission, 590 nm) was measured using Biotek Synergy H4 Multi-Mode Plate Reader. Each sample was measured in triplicate. For cAMP content measurements, treated zebrafish larvae were collected (10 larvae were pooled per condition), homogenized in 100 μL 0.1 M HCl, and centrifuged at 6,000 *g* for 10 min at 4°C. The rat INS-1 cells were cultured, serum-starved and chemical-treated as described above. After treatment, cells were lysed in 200 μL 0.1 M HCl and centrifuged. Supernatants from all samples were collected and stored at −80°C until cAMP content measurements.

Mammalian *ex vivo* islet culture

Male Lewis rats were housed on a 12-hour light/dark cycle in a controlled climate according to Georgia Institute of Technology regulations under Institutional Animal Care and Use Committee-approved protocols. Pancreatic islets were isolated from rats by collagenase digestion (Liberase TL, Roche Diagnostics) and gradient purification, and then hand-picked and pooled. Islets were cultured overnight in CMRL-1066 (Invitrogen) supplemented with 10% FBS, 1%

penicillin/streptomycin, and 25 mM HEPES (Invitrogen). The next day, islets were either directly treated with 3-(3-phenylbenzo[c]isoxazol-5-yl)acrylic acid (PIAA) for 4 days or dissociated with 0.05% trypsin-EDTA (Invitrogen) for 3-5 minutes with gentle agitation to aid cell cluster disruption. Single cells obtained from islet dissociation were plated in 96-well plates and allowed 48 hours to attach prior to treatment with 3-(3-phenylbenzo[c]isoxazol-5-yl)acrylic acid (PIAA) for 4 days. Human islets were cultured in Human Islet Media (Prodo Labs) supplemented with 1% penicillin/streptomycin and 10% human serum for 24-48 hours prior to experimentation.

Glucose-stimulated insulin secretion from *ex vivo* islet culture

Human and rat islets were evaluated for glucose-stimulated insulin secretion using standard methods. Cultured islets with 4-5 days treatment with DMSO or 40-80 μM PIAA were equilibrated within a column in the center of a 1 mL sandwich of Sephadex beads using 3 mM glucose in Krebs buffer for one hour. After equilibration, 3 replicates per group were subjected to 1 hour sequential incubations with Krebs low (3 mM glucose), high (16 mM glucose), and low again. Secreted insulin was monitored using a human or rat ELISA kit (Mercodia) and normalized to total DNA (PicoGreen, Invitrogen).

Mice experiments

Only mice that had fed blood glucose values of >300 mg glucose/dL were used. Mice were given daily intraperitoneal injections of vehicle (dimethylsulphoxide final 6.7%; formulated in 0.5% methylcellulose and 0.5% Tween-80) or PIAA (12.5 mg per kg body weight; formulated in 0.5%

methylcellulose and 0.5% Tween-80). Mice were housed in pathogen-free facilities and maintained in the Animal Care Facilities at Mayo Clinic, Rochester, MN and Parker H. Petit Institute for Bioengineering and Bioscience, Georgia Institute of Technology, Atlanta, GA.

Glucose tolerance test

For glucose tolerance test, after a 12-hour fast, mice were given an intraperitoneal injection of glucose at a dose of 1-2 g per kg body weight. For insulin tolerance test, after a 6-hour fast, 0.75 mU/g body weight of insulin was given. We measured blood glucose at basal, 30, 60, 90, and 120 minutes from tail blood using the OneTouch Ultra glucometer (Lifescan).

Assessment of insulin content

Isolated mice pancreata were placed in acid-ethanol mixture (0.18 M HCl, 75% EtOH), incubated overnight (−80°C), and then homogenized; the tissue levels of insulin extracted by centrifugation (2,000 rpm, 15 min, 4 °C) were then determined by mouse insulin ELISA kit (Mercodia) and normalized to total pancreatic protein content determined by Pierce BCA protein assay kit (Thermo Fisher).

Ki67-postive cell counting

INS-1 cells

To calculate the number of proliferating β-cells, an image of an area (near the center of each well) was taken using a Zeiss LSM 700-405 confocal microscope (10X, zoom 0.5; Image size

1280 μM*1280 μM). The number of Ki67+ and Insulin+ double positive cells, and the total number of Insulin+ cells in the area were assessed with ImageJ.

Rat islets

To calculate the number of proliferating β-cells in cultured whole rat islets, an image of an area (near the edge of each islet) with Insulin+ cells was taken using a Zeiss LSM 700-405 confocal microscope (20X, zoom 0.5; Image size 640 μM*640 μM) The number of Ki67+ and Insulin+ double positive cells, and the total number of Insulin+ cells were counted with ImageJ (20 planes of confocal images). 5 islets per treatment were counted.

Human islets

To calculate the number of proliferating β-cells in dissociated cultured human islets, the total number of Ki67+ and INSULIN+ double positive cells in each well was manually counted using a Zeiss LSM 700-405 confocal microscope. The total number of INSULIN+ cells in each well was estimated approximately by multiplying the number of INSULIN+ cells in a single cluster of islet and the total number of clusters with INSULIN+ cells.

STZ-induced diabetic mice

To calculate the number of proliferating β-cells, images of >10 islets in two 8 μm-thick pancreas sections (separated by 400 μm) per animal were taken using a Zeiss LSM 700-405 confocal microscope. The number of Ki67+ and Insulin+ double positive cells, and the total number of Insulin+ cells in the same islet were counted with ImageJ (20 planes of confocal images; n=6 mice per group). For the table 4, representative 12 islets were shown.

Synthesis and chemical properties of PIAA

Chemistry

Compounds **1** (**PIAA1**), **2** (**PIAA 2**), **4** (**PIAA**), **5** (**PIAA3**) and **6** (**PIAA4**) were made from commercially available *p*-bromonitrobenzene (Figure S1A). Following a known literature procedure[3], *p*-bromonitrobenzene was converted to 5-bromo-3-phenylbenzo[*c*]isoxazole (compound **1, PIAA1**). Heck coupling reaction between compound **1** and methyl acrylate gave the benzoxazole derivative (compound **2, PIAA2**) along with a by-product whose ^{1}H NMR matched with the structure of the compound **3**. Finally, these methyl ester compounds **2** and **3** were subjected to saponification reaction to give the target molecules compound **4** (**PIAA**) and compound **5** (**PIAA3**). Heck coupling reaction between compound **1** and acrylamide gave the target compound **6**.

Synthesis of compound **11** (***iso*-PIAA**) was achieved starting from commercially available *m*-bromoaniline (Figure S1B). Following a known literature procedure[4], *m*-bromoaniline was converted to a substituted aminobenzophenone compound **7**. Transformation of the amino group of compound **7** to an azido group followed by Fe(II)-catalyzed[5] intramolecular N–O bond formation yielded the 6-bromo-3-phenylbenzo[*c*]isoxazole (compound **8)**. Pd(II)-catalyzed Heck coupling[6] of compound **8** with methyl acrylate gave the benzisoxazole derivative (compound **9)** and a byproduct whose ^{1}H NMR matched the structure of compound **10**. Saponification of compound **9** gave the target molecule (compound **11**, ***iso*-PIAA**).

General experimental procedures

All commercially available starting materials were used without further purification. Reaction solvents were either high performance liquid chromatography (HPLC) grade or American Chemical Society (ACS) grade and used without further purification. Analtech silica gel plates (60

F254) were used for analytical TLC and Analtech preparative TLC plates (UV 254, 2000 μm) were used for purification. UV light, and anisaldehyde and iodine stains were used to visualize the spots. 100-200 Mesh silica gel was used in column chromatography. Nuclear magnetic resonance (NMR) spectra were recorded on a Varian-Gemini 400 MHz or Bruker 500 MHz magnetic resonance spectrometer. ^{1}H NMR spectra were recorded in parts per million (ppm) relative to the residual peaks of $CHCl_3$ (7.24 ppm), CHD_2OD (4.78 ppm) or DMSO-d_5 (2.49 ppm). ^{13}C spectra were recorded relative to the central peak of the $CDCl_3$ triplet (77.0 ppm) or CD_3OD septet (49.3 ppm) or DMSO-d_6 septet (39.7 ppm) and were recorded with complete hetero-decoupling. High-resolution mass spectra were recorded at the Georgia Institute of Technology mass spectrometry facility.

Methyl (*E*)-3-(3-phenylbenzo[*c*]isoxazole-5yl)acrylate (compound **2, PIAA2)**. A solution of 5-bromo-3-phenylbenzo[*c*]isoxazole (compound **1**; 215 mg, 0.78 mmol), methyl acrylate (0.14 mL, 0.57 mmol), palladium(II) acetate (26 mg, 0.12 mmol), tri-*o*-tolylphosphine (74 mg, 0.24 mmol), and trimethylamine (0.27 mL, 1.96 mmol) in degassed acetonitrile (3 mL) was heated at 90 °C in a sealed pressure tube for 6 h. Subsequently, the reaction mixture was cooled to room temperature, diluted with ethyl acetate (100 mL), and washed with 4M HCl (20 mL), water (20 mL), and brine (10 mL). The ethyl acetate portion was dried over anhydrous Na_2SO_4, filtered, and concentrated in *vacuo*. The crude product was purified by column chromatography (SiO_2, 20% ethyl acetate in hexane) to afford the pure title compound **2** (**PIAA2**, 68 mg, 31%) as a yellow solid. ^{1}H NMR (400 MHz, DMSO-d_6) δ 8.49 (s, 1H), 8.18 (d, J = 7.2 Hz, 2H), 7.84 (t, J = 13.2 Hz, 2H), 7.72 – 7.55 (m, 4H), 6.72 (d, J = 16.1 Hz, 1H), 3.75 (s, 3H).

Methyl (*E*)-3-(4-amino-3-benzophenyl)acrylate (compound **3**). During the synthesis of the compound **2** (**PIAA2**), another by-product was detected by TLC and was isolated by column chromatography (SiO_2, 50% ethyl acetate in hexane) and characterized as compound **3** (yellow solid, 142 mg, 65%). ^{1}H NMR (400 MHz, DMSO-d_6) δ 7.76 – 7.64 (m, 1H), 7.62 – 7.45 (m, 6H), 7.39 (d, J = 15.7 Hz, 1H), 7.35 (d, J = 15.8 Hz, 1H), 7.25 (s, 1H), 6.89 (d, J = 8.8 Hz, 1H), 6.17 (d, J = 15.9 Hz, 1H), 3.63 (s, 3H). HRMS (EI) *m/z* Calcd. for $C_{17}H_{15}ON_3$ [M^+]: 281.1052, found 281.1040.

(*E*)-3-(3-phenylbenzo[*c*]isoxazole-5yl)acrylic acid **(**compound **4, PIAA)**. Compound **2** (**PIAA2**, 60 mg, 0.21 mmol) was dissolved in THF-MeOH-H_2O (2 mL, 3:1:1) and LiOH·H_2O (18 mg, 0.42 mmol) was added to the solution. The resulting mixture was stirred at room temperature for 3 h. 1M HCl was added to the reaction mixture to quench the reaction and pH of the solution was brought down to 2. The resulting solution was extracted with 10% MeOH in dichloromethane (50 mL) and washed with water (10 mL) and brine (10 mL). The organic layer was dried over anhydrous Na_2SO_4, filtered, and concentrated in *vacuo*. The crude product was triturated with dichloromethane to give the title compound **4** (**PIAA**, 37 mg, 66%) as a yellow solid. ^{1}H NMR (400 MHz, DMSO-d_6) δ 12.43 (s, 1H), 8.45 (s, 1H), 8.18 (dd, J = 8.1, 1.4 Hz, 2H), 7.86 (d, J = 9.4 Hz, 1H), 7.82 – 7.52 (m, 5H), 6.62 (d, J = 16.0 Hz, 1H). ^{13}C NMR (101 MHz, DMSO-d_6) δ 167.8, 165.3, 157.4, 143.4, 131.8, 131.3, 129.8, 127.3, 126.9, 124.2, 119.9, 115.8, 114.2. HRMS (ESI) *m/z* Calcd. for $C_{16}H_{10}O_3N$ [M – H^+]: 264.0666, found 264.0669.

(*E*)-3-(4-amino-3-benzophenyl)acrylic acid (compound **5**, **PIAA3)**. Following the same procedure as described for the synthesis of compound **4** (**PIAA)**, saponification of the compound **3** (130 mg,

0.46 mmol) with LiOH·H_2O (39 mg, 0.92 mmol) in THF-MeOH-H_2O (2 mL, 3:1:1) gave the title compound **5** (**PIAA3**, 119 mg, 80%) as a yellow solid. ^{1}H NMR (400 MHz, DMSO-*d*$_6$) δ 12.01 (s, 1H), 7.69 (d, *J* = 9.1 Hz, 1H), 7.67 – 7.41 (m, 7H), 7.33 (d, *J* = 15.9 Hz, 2H), 6.89 (d, *J* = 9.0 Hz, 1H), 6.10 (d, *J* = 15.8 Hz, 1H). HRMS (ESI) *m/z* Calcd. for $C_{16}H_{12}O_3N$ [M – H$^+$]: 266.0823, found 266.0823.

(E)-3-(3-phenylbenzo[c]isoxazole-5-yl)acrylamide (compound **6**). Heck coupling reaction between compound **1** (75 mg, 0.27 mmol) and acrylamide (39 mg, 054 mmol) in presence of Pd(OAc)$_2$ (9.1 mg, 0.04 mmol) gave the title compound **6** (4 mg, 5%) as yellow solid. ^{1}H NMR (400 MHz, DMSO-*d*$_6$) δ 8.35 (s, 1H), 8.18 (d, *J* = 6.8 Hz, 2H), 7.78 – 7.51 (m, 7H), 7.16 (s, 1H), 6.68 (d, *J* = 16.0 Hz, 1H). HRMS (ESI) *m/z* Calcd. for $C_{16}H_{13}O_2N_2$ [M + H]: 265.0972, found 265.0978.

6-Bromo-3-phenylbenzo[*c*]isoxazole (compound **8**). Sodium nitrite (103 mg, 1.49 mmol) was added to a solution of substituted aminobenzophenone derivative **7** (300 mg, 0.99 mmol) in 5 mL of AcOH and 5 mL of H_2O at 0 °C. After 1 h of stirring at the same temperature, sodium azide (96 mg, 1.49 mmol) was added and the resulting mixture was stirred at room temperature for 2 h. Reaction mixture was neutralized with a saturated aqueous Na_2CO_3 solution and was extracted with dichloromethane (50 mL). The organic layer was washed with water (20 mL) and brine (20 mL), dried over anhydrous Na_2SO_4, filtered, and concentrated in *vacuo*. The crude product was used in the next step without further characterization. The crude aryl azide was mixed with dried 4Å molecular sieve (300 mg, 100 mol%), $FeBr_2$ (11mg, 0.05 mmol), and anhydrous dichloromethane (1 mL) in a sealed tube and the resultant mixture was heated at 40 °C for 16 h.

Subsequently, the mixture was cooled and filtered. The filtrate was concentrated and the crude product was purified by column chromatography (SiO_2, 20% ethyl acetate in hexane) to give the title compound **8** (163 mg, 60%) as a white solid. ^{1}H NMR (400 MHz, $CDCl_3$) δ 7.98 (d, J = 6.8 Hz, 2H), 7.83 (s, 1H), 7.72 (d, J = 9.2 Hz, 1H), 7.59 – 7.51 (m, 4H). HRMS (EI) *m/z* Calcd. for $C_{13}H_8ONBr$ [M^+]: 272.9788, found 272.9789.

Methyl (*E*)-3-(3-phenylbenzo[*c*]isoxazole-6yl)acrylate (compound **9)**. A solution of 6-bromo-3-phenylbenzo[*c*]isoxazole (compound **8**; 184 mg, 0.67 mmol), methyl acrylate (0.12 mL, 1.34 mmol), palladium(II) acetate (23 mg, 0.10 mmol), tri-*o*-tolylphosphine (63 mg, 0.20 mmol), and triethylamine (0.23 mL, 1.68 mmol) in degassed acetonitrile (2 mL) was heated at 90 °C in a sealed pressure tube for 6 h. Subsequently, the reaction mixture was cooled to room temperature, diluted with ethyl acetate (100 mL), and washed with 4M HCl (20 mL), water (20 mL), and brine (10 mL). The ethyl acetate portion was dried over anhydrous Na_2SO_4, filtered, and concentrated in *vacuo*. The crude was purified by column chromatography (SiO_2, 20% ethyl acetate in hexane) to afford the pure title compound **9** (24 mg, 13%) as a yellow solid. ^{1}H NMR (500 MHz, DMSO-d_6) δ 8.12 (dt, J = 12.9, 4.8 Hz, 3H), 8.03 (s, 1H), 7.78 (d, J = 16.1 Hz, 1H), 7.69 – 7.55 (m, 4H), 6.85 (d, J = 16.1 Hz, 1H), 3.76 (s, 3H). HRMS (ESI) *m/z* Calcd. for $C_{17}H_{14}O_3N$ [$M + H^+$]: 280.0968, found 280.0969.

Methyl (*E*)-3-(3-amino-4-benzophenyl)acrylate (compound **10)**. The title compound **10** was obtained as a byproduct during the Heck coupling reaction between 6-bromo-3-phenylbenzo[*c*]isoxazole (compound **8**; 184 mg, 0.67 mmol) and methyl acrylate (0.12 mL, 1.34 mmol). The yield was 73% (137 mg) [eluted with 30% ethyl acetate in hexane]. ^{1}H NMR (500

MHz, DMSO-d_6) δ 7.65 – 7.45 (m, 6H), 7.26 (dd, J = 20.7, 8.0 Hz, 1H), 7.16 (s, 2H), 7.07 (d, J = 1.3 Hz, 1H), 6.93 – 6.84 (m, 1H), 6.58 (d, J = 16.0 Hz, 1H), 3.73 (s, 3H). HRMS (EI) *m/z* Calcd. for $C_{17}H_{15}ON_3$ [M^+]: 281.1052, found 281.1038.

(*E*)-3-(3-phenylbenzo[*c*]isoxazole-6yl)acrylic acid (compound **11**, *iso*-**PIAA)**. Compound **9** (20 mg, 0.07 mmol) was dissolved in 2 mL of THF-MeOH-H_2O (3:1:1) and LiOH·H_2O (6.10 mg, 0.14 mmol) was added to the solution. The resulting mixture was stirred at room temperature for 3 h. 1M HCl was added to the reaction mixture to quench the reaction and pH of the solution was brought down to 2. The resulting solution was extracted with 10% MeOH in dichloromethane (50 mL) and was washed with water (10 mL) and brine (10 mL). The organic layer was dried over anhydrous Na_2SO_4, filtered, and concentrated in *vacuo*. The crude product was triturated with dichloromethane to give the pure title compound **11** (***iso*-PIAA**, 12 mg, 70%) was as a yellow solid. ^{1}H NMR (500 MHz, DMSO-d_6) δ 12.60 (s, 1H), 8.11 (dd, J = 13.3, 5.8 Hz, 3H), 7.97 (d, J = 24.6 Hz, 1H), 7.71 (d, J = 16.0 Hz, 1H), 7.68 – 7.61 (m, 2H), 7.62 – 7.54 (m, 2H), 6.81 – 6.66 (m, 1H). ^{13}C NMR (101 MHz, DMSO-d_6) δ 167.2, 164.1, 157.6, 142.6, 137.2, 130.7, 129.6, 126.8, 123.1, 122.2, 121.3, 116.9, 113.7. HRMS (ESI) *m/z* Calcd. for $C_{16}H_{10}O_3N$ [$M - H^+$]: 264.0666, found 264.0667.

Docking simulations

In silico docking was performed using Autodock Vina[7] run through PyRx to manage the workflow and PyMol to visualize the results. Ligands were prepared by generating an energy minimized 3D structure in ChemBioDraw3D. Then the energy minimized ligands and macromolecule (TBK1 (PDB code: 4EUT)) were processed with Autodock Tools 1.5.4. Docking

runs were performed within a 25−30 Å cubic search space surrounding the TBK1 binding pocket previously occupied by BX795. After the run, Autodock Vina ranked docking outputs according to binding affinity. Docking outputs were thereafter overlaid with BX795 in the TBK1 binding pocket to select the output that made the appropriate interactions with residues in the binding pocket.

Elucidation of the Structure of PIAA

PIAA was originally described as (E)-3-(3-phenylbenzo[c]isoxazol-6-yl)acrylic acid (*iso*-PIAA). However, a comparison of its docking pose with that of (E)-3-(3-phenylbenzo[c]isoxazol-5-yl)acrylic acid (compound **4**) showed that compound **4** is better accommodated at the active site of TBK1 than *iso*-PIAA (Figure 2C). The β-cell regeneration activity of compound **4** is indistinguishable from that of ChemDiv *compound 7366-0123*, the compound identified from ChemDiv screening collection as TBK1 inhibitor[8], while *iso*-PIAA is inactive (data not shown). Moreover, compound **4** inhibits TBK1/IKKε kinase activity while *iso*-PIAA is inactive (Figures 2A-2B). Finally, the ^{1}H-NMR of compound **4** matched the ^{1}H-NMR provided by ChemDiv. These data confirmed that the correct structure of PIAA is (E)-3-(3-phenylbenzo[c]isoxazol-5-yl)acrylic acid (compound **4**).

Supplemental References

1 Bajoghli, B., Aghaallaei, N., Heimbucher, T. & Czerny, T. An artificial promoter construct for heat-inducible misexpression during fish embryogenesis. *Dev Biol* **271**, 416-430, doi:10.1016/j.ydbio.2004.04.006 (2004).

2 Kawakami, K., Koga, A., Hori, H. & Shima, A. Excision of the tol2 transposable element of the medaka fish, Oryzias latipes, in zebrafish, Danio rerio. *Gene* **225**, 17-22 (1998).
3 Orlov, V. Y., Kotov, A. D., Tsivov, A. V. & Rusakov, A. I. Mechanism of formation of 2,1-benzisoxazoles in reactions of nitroarenes with arylacetonitriles. *Russian Journal of Organic Chemistry* **51**, 245-252, doi:10.1134/s1070428015020190 (2015).
4 Andres, J. I. *et al.* Synthesis and structure-activity relationship of 2-(aminoalkyl)-2,3,3a,8-tetrahydrodibenzo[c,f]isoxazolo[2,3-a]azepine derivatives: a novel series of 5-HT(2A/2C) receptor antagonists. Part 2. *Bioorganic & medicinal chemistry letters* **12**, 249-253 (2002).
5 Stokes, B. J., Vogel, C. V., Urnezis, L. K., Pan, M. & Driver, T. G. Intramolecular Fe(II)-Catalyzed N−O or N−N Bond Formation from Aryl Azides. *Organic letters* **12**, 2884-2887, doi:10.1021/ol101040p (2010).
6 Whitcombe, N. J., Hii, K. K. & Gibson, S. E. Advances in the Heck chemistry of aryl bromides and chlorides. *TETRAHEDRON* **57**, 7449-7476, doi:10.1016/S0040-4020(01)00665-2 (2001).
7 Trott, O. & Olson, A. J. AutoDock Vina: improving the speed and accuracy of docking with a new scoring function, efficient optimization, and multithreading. *Journal of computational chemistry* **31**, 455-461, doi:10.1002/jcc.21334 (2010).
8 Hutti, J. E. *et al.* Development of a high-throughput assay for identifying inhibitors of TBK1 and IKKepsilon. *PLoS One* **7**, e41494, doi:10.1371/journal.pone.0041494 (2012).

Supplementary table 1. Quantification of Insulin- and Ki67/Insulin-double positive cell number in INS-1 cells

		Fields	Ki67+/Ins+	Insulin+	%
INS-1	DMSO	1	35	93	37.63
		2	73	152	48.03
		3	55	126	43.65
	PIAA	1	158	162	97.53
		2	248	263	94.3
		3	173	211	81.99
		4	172	228	75.44

Supplementary table 2. Quantification of Insulin- and Ki67/Insulin-double positive cell number in rat islets

		Islets	Ki67+/Ins+	Insulin+	%
**Rat**	**DMSO**	1	2	210	0.95
		2	3	250	1.20
		3	2	240	0.83
		4	2	220	0.91
		5	4	240	1.67
	**PIAA 20 µM**	1	8	200	4.00
		2	3	180	1.67
		3	4	220	1.82
		4	6	250	2.40
		5	7	190	3.68
	**PIAA 40 µM**	1	10	200	5.00
		2	11	230	4.78
		3	5	270	1.85
		4	3	180	1.67
		5	9	180	5.00
	**PIAA 80 µM**	1	10	180	5.56
		2	12	200	6.00
		3	10	160	6.25
		4	9	190	4.74
		5	7	140	5.00

Supplementary table 3. Quantification of Insulin- and Ki67/Insulin-double positive cell number in dissociated human islet culture

		Wells	Ki67+/INS+	INSULIN+	%
**Human**	**DMSO**	1	2	1385	0.14
		2	2	1450	0.14
		3	3	1500	0.20
		4	1	1210	0.08
		5	1	1100	0.09
	**PIAA 20 µM**	1	6	1510	0.40
		2	4	1350	0.30
		3	2	1410	0.14
		4	4	1400	0.29
		5	4	1460	0.27
	**PIAA 40 µM**	1	7	1425	0.49
		2	4	1685	0.24
		3	5	1380	0.36
		4	6	1450	0.41
		5	5	1520	0.33
	**PIAA 80 µM**	1	12	1545	0.78
		2	7	1400	0.50
		3	5	1450	0.34
		4	8	1350	0.59
		5	5	1650	0.30

Supplementary table 4. Quantification of Insulin- and Ki67/Insulin-double positive cell number in mice pancreata

		Islets	Ki67+/Ins+	Insulin+	%
STZ mice	DMSO	1	0	48	0
		2	0	58	0
		3	0	79	0
		4	1	65	1.54
		5	0	55	0
		6	0	62	0
		7	1	143	0.7
		8	1	42	2.38
		9	1	102	0.98
		10	1	42	2.38
		11	0	68	0
		12	0	31	0
	PIAA	1	1	62	1.61
		2	3	52	5.77
		3	2	64	3.13
		4	4	130	3.08
		5	2	87	2.3
		6	2	65	3.08
		7	2	96	2.08
		8	3	84	3.57
		9	4	108	3.7
		10	3	80	3.75
		11	2	141	1.42
		12	4	126	3.18

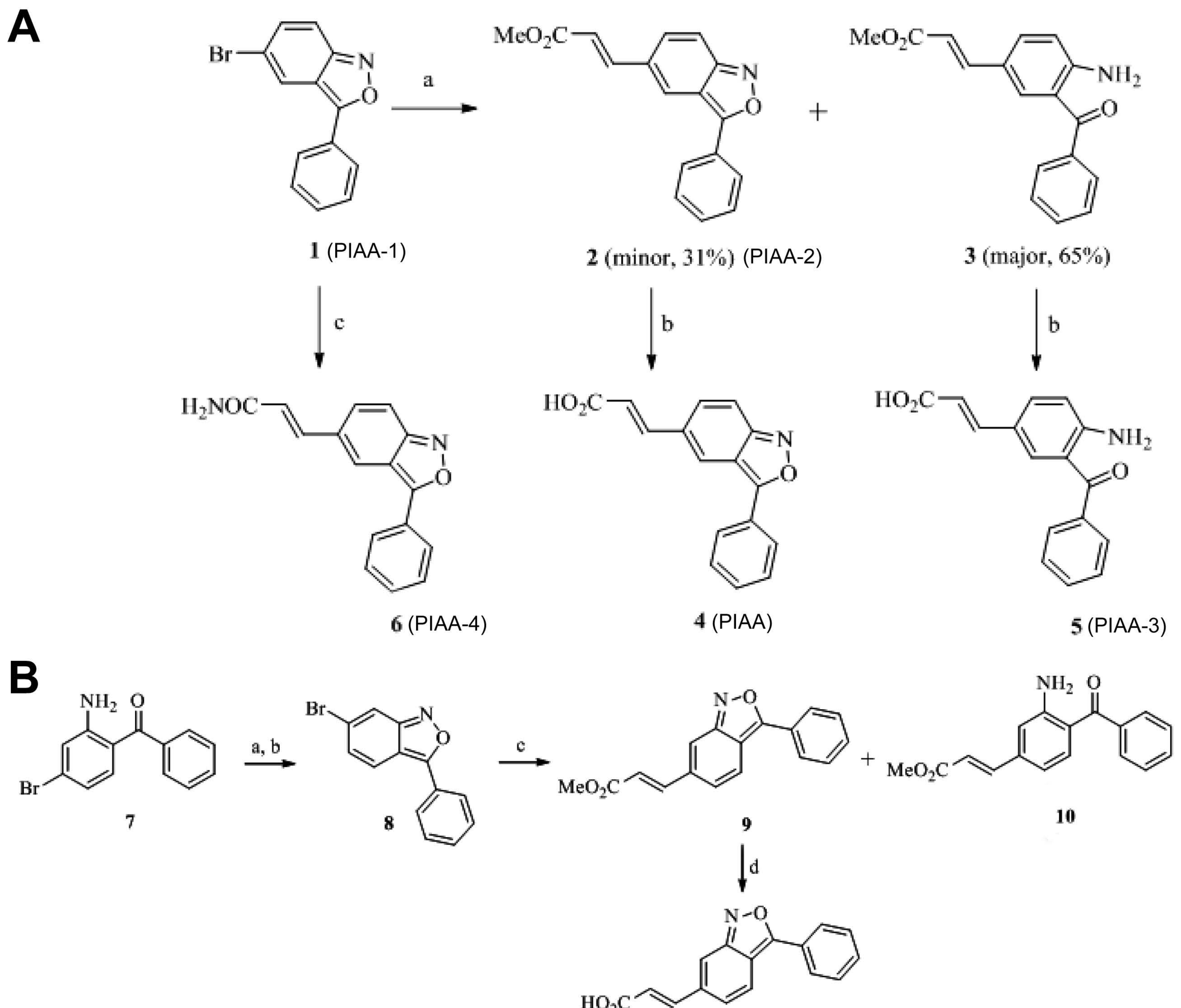

Supplementary Figure 1

J

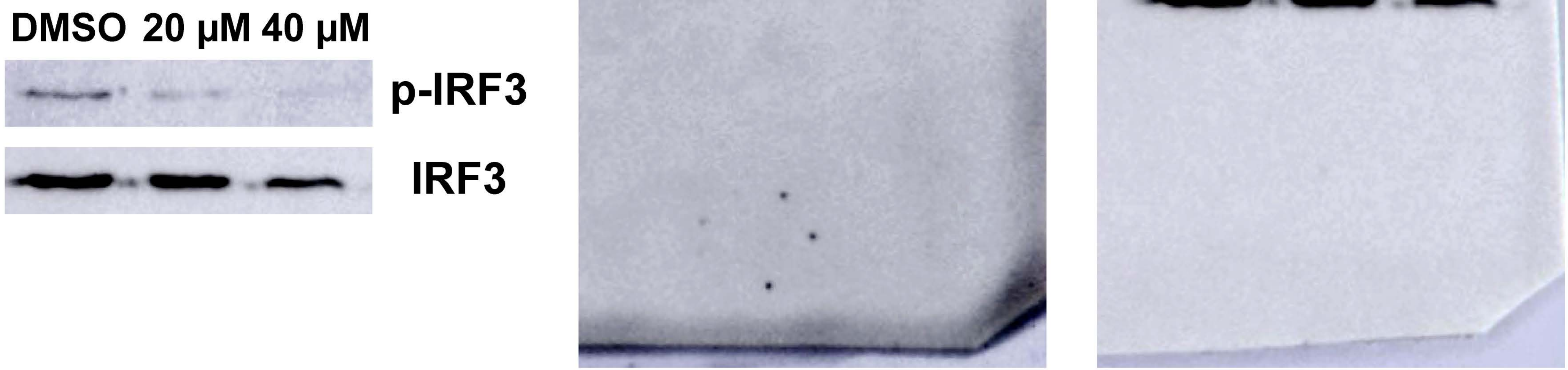

Supplementary Figure 2

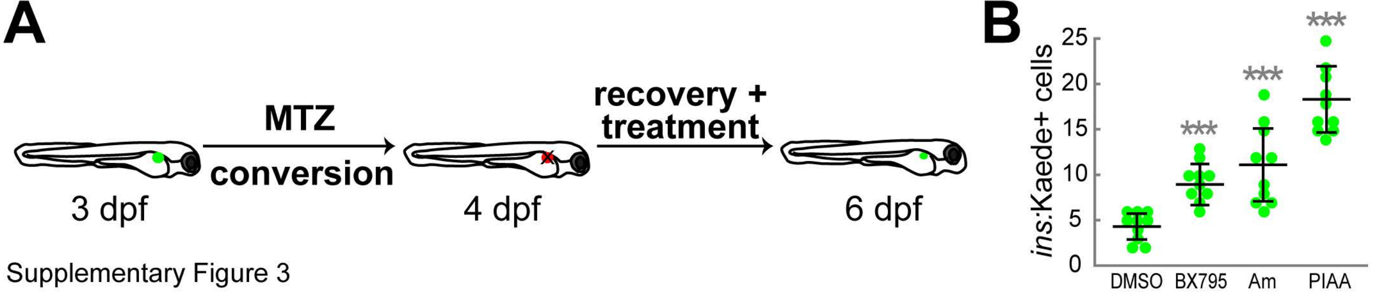

Supplementary Figure 3

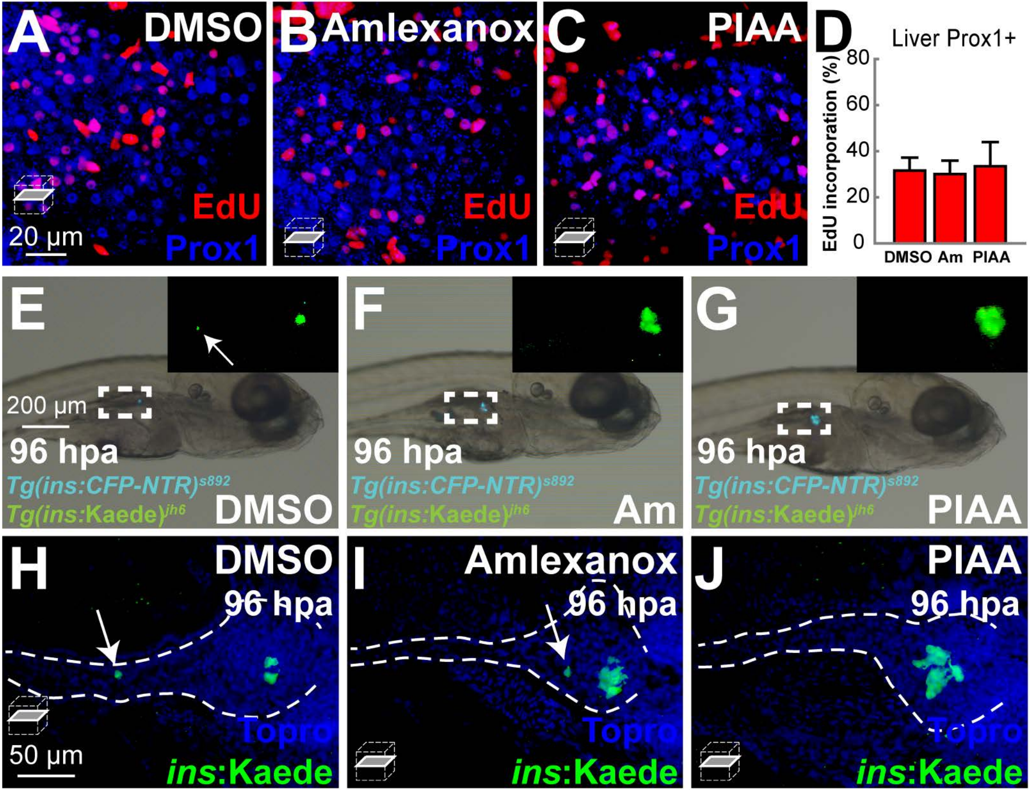

Supplementary Figure 4

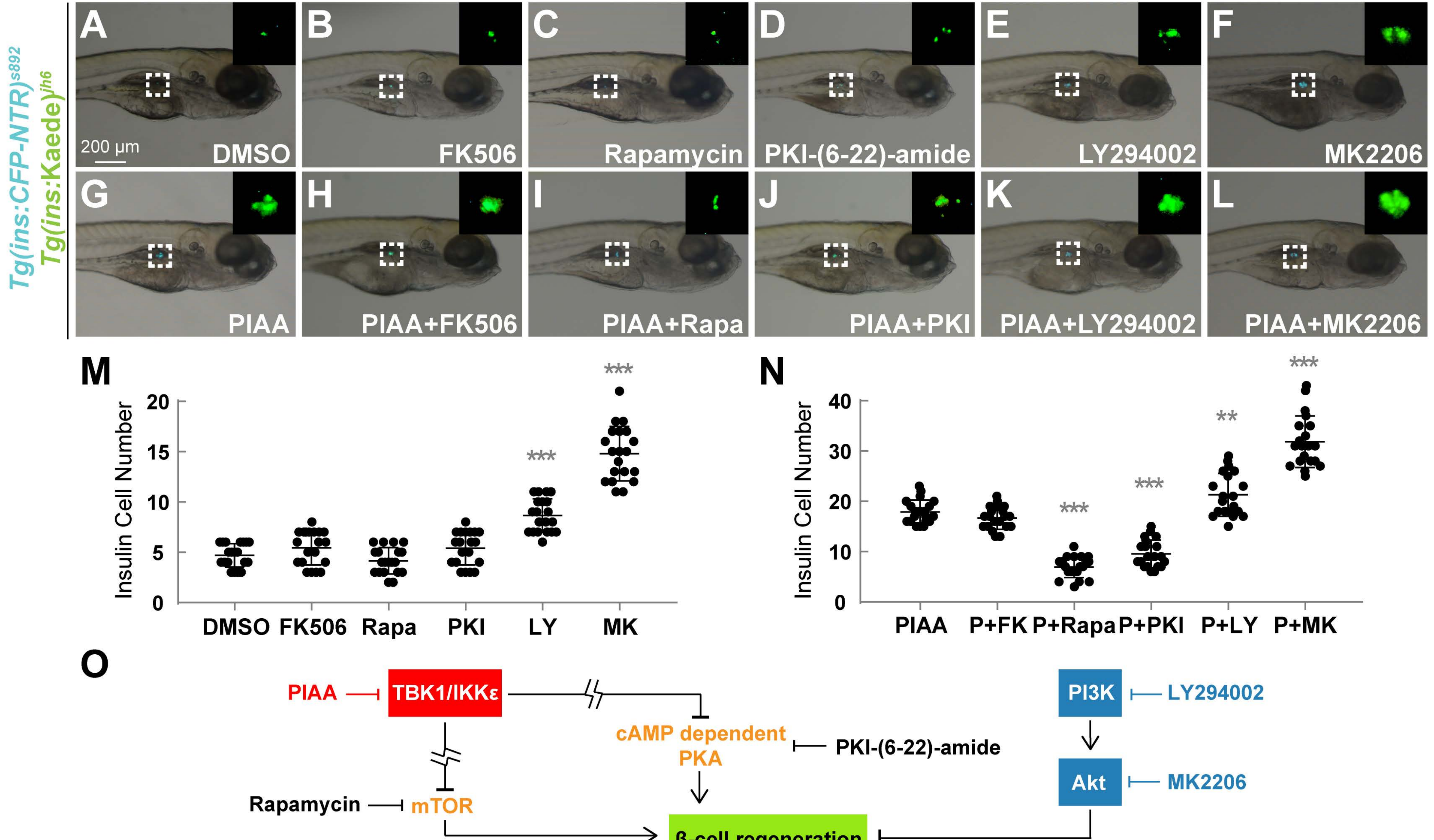

Supplementary Figure 5

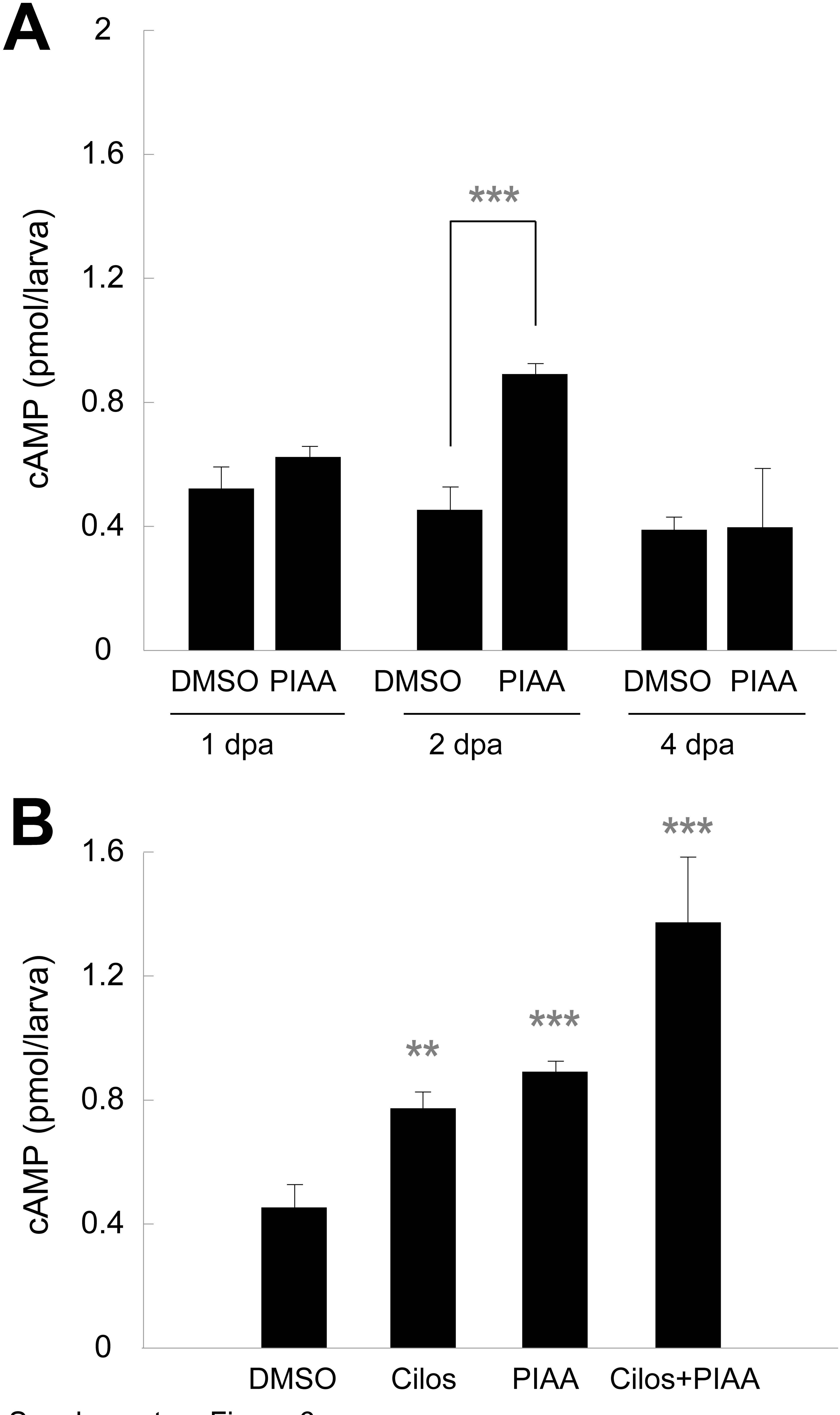

Supplementary Figure 6

C

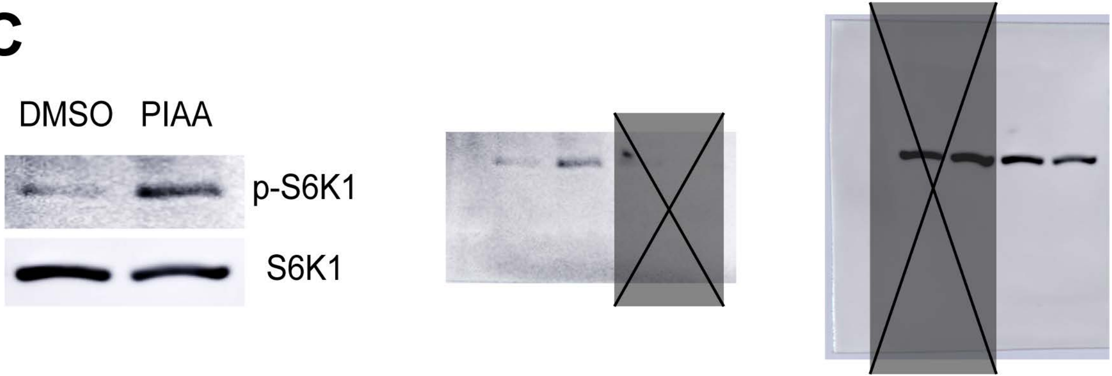

Supplementary Figure 7

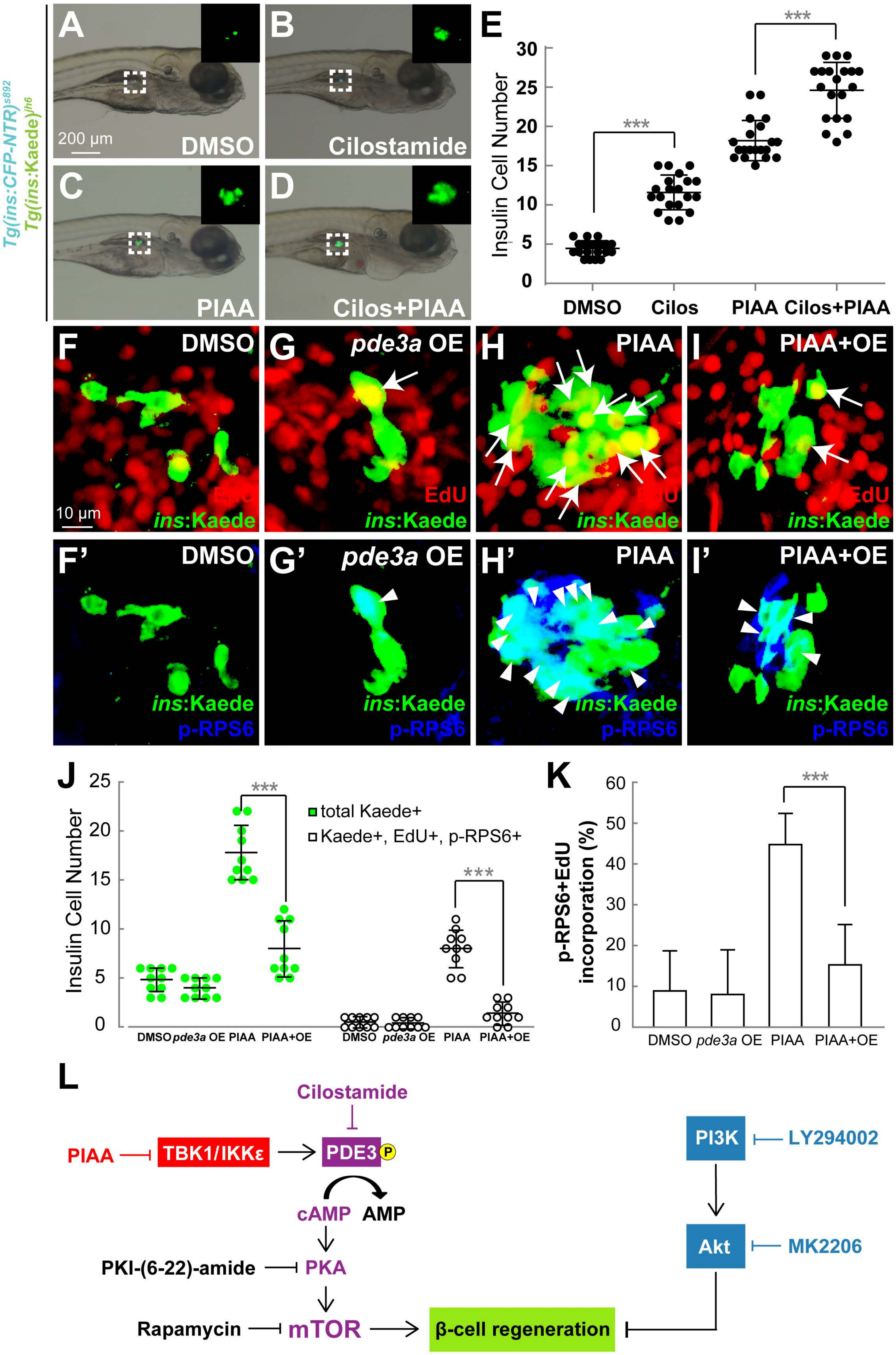

Supplementary Figure 8

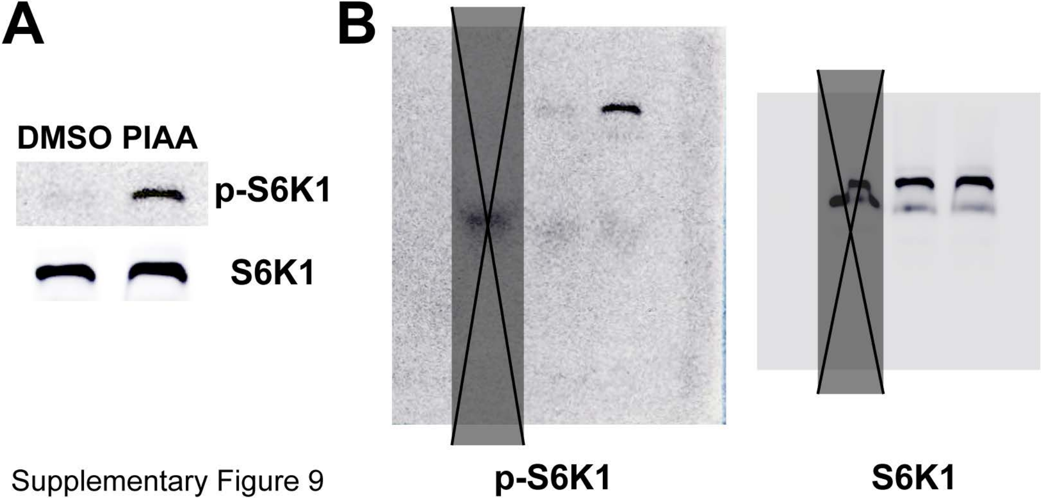

Supplementary Figure 9

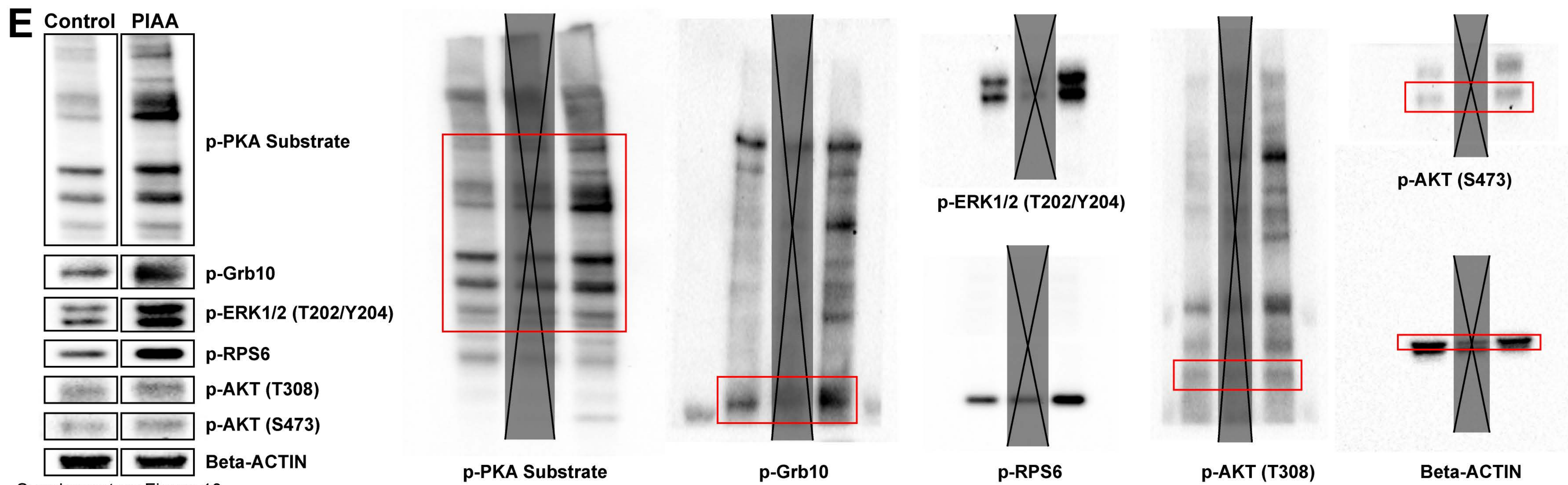

Supplementary Figure 10

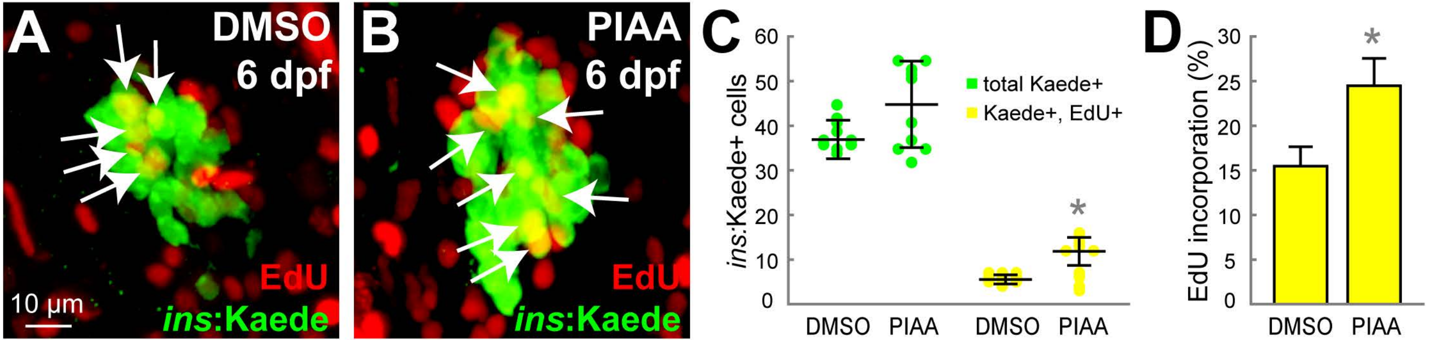

Supplementary Figure 11
